# Carbon-Core/Molecular-State-Regulated Red/Blue Dual-Emission Carbon Quantum Dots Covalently Anchored on Polyvinyl Alcohol for Multifunctional Agricultural Films in Greenhouse Potato Production

**DOI:** 10.3390/polym18121442

**Published:** 2026-06-09

**Authors:** Zhimin Ye, Jiwei Liu, Maolin Wang, Kun Huang, Li Zhang, Yuanyuan Jiang, Ying Wang, Yunsong Zhang, Li Lin

**Affiliations:** 1 College of Science, Sichuan Agricultural University, Ya’an 625014, China; yzm13398308553@163.com (Z.Y.); 6jvliu@gmail.com (J.L.); maolin1217@126.com (M.W.); zhangli@sicau.edu.cn (L.Z.); yyjiang607@sina.cn (Y.J.); 2 National Engineering Research Center of Clean Technology in Leather Industry, Sichuan University, Chengdu 610065, China; gmzc2026@163.com; 3 College of Water Conservancy and Hydropower Engineering, Sichuan Agricultural University, Ya’an 625014, China; yw1981@gmail.com

**Keywords:** dual-emission carbon quantum dots, covalent anchoring, multifunctional agricultural films, self-cleaning, photosynthesis enhancement

## Abstract

For agricultural films, spectral matching, UV protection, and environmental durability are essential for efficient crop production. A self-cleaning silane-crosslinked red/blue dual-emission carbon dot/polyvinyl alcohol composite film (KH/RB-CQDs/PVA) was fabricated via a covalent anchoring strategy. RB-CQDs were synthesized by a two-step hydrothermal method using o-phenylenediamine: initial blue-emitting carbon cores formed, then phosphoric acid-assisted secondary treatment covalently bridged residual precursor-derived red fluorophores onto cores through pyrophosphate bonds, as evidenced by TEM, XPS, ^31^P NMR, HPLC-MS and DFT. This rigid bridging suppressed excessive core growth and energy transfer while spatially separating dual emission, endowing excellent photostability (>95% fluorescence retention after 50 min UV and 30 d storage). Subsequently, KH-560 was employed to construct a robust covalent crosslinked network anchoring RB-CQDs in PVA and forming rough Si-O-Si surface structures, confirmed by SEM and XPS. The resulting film exhibited 16.16% quantum yield, 291% tensile strength enhancement, 95% UV shielding, and <1% contaminant residue. Chlorophyll fluorescence kinetics, gas-exchange analyses, and photosynthetic response curves demonstrated that KH/RB-CQDs/PVA increased the potato net photosynthetic rate by 55.46% and tuber yield by 76% through synergistic optimization of photosystem II electron transport and RuBisCO-mediated carbon assimilation. This work provides a molecular design principle for high-performance intelligent agricultural films.

## 1. Introduction

Greenhouses serve as pivotal platforms for enhancing both yield and efficiency in modern agriculture, as the material properties of covering films directly determine crop light-use efficiency and facility durability [1,2]. However, current agricultural films face three critical bottlenecks: (i) conventional covering materials lack precise spectral matching with solar irradiation, failing to meet the specific light-quality requirements for crop photosynthesis [3]; (ii) sustained ultraviolet (UV) exposure induces photooxidative degradation of the polymer matrix, resulting in yellowing, embrittlement, and shortened service life [4,5]; and (iii) progressive accumulation of surface contaminants leads to a continuous decline in light transmittance [6]. To address these challenges, we have developed a multifunctional light-conversion film based on a covalent anchoring strategy that integrates three innovations not previously combined in a single system: a pyrophosphate-bridged carbon-core/molecular-state dual-emission architecture for stable red/blue spectral conversion, a silane-crosslinked network for mechanical robustness and self-cleaning surface hydrophobicity, and systematic in vivo validation of photosynthetic enhancement. This work thus provides a molecular design principle for next-generation high-performance greenhouse covering materials [7].

Carbon quantum dots (CQDs) have emerged as promising light-conversion materials for agricultural applications owing to their excellent ultraviolet (UV) conversion capability, low cost, and favorable biocompatibility [8]. Previous studies have shown that blue-emissive CQDs can significantly promote biomass accumulation in rice and lettuce [9,10], whereas red light plays a central role in photomorphogenesis and carbon metabolism [11]. Beyond light conversion, carbon nanomaterials can also serve as bioimaging probes for real-time monitoring of plant physiological processes [12]. Importantly, the combination of red and blue spectra can synergistically enhance photosynthetic efficiency, stimulating extensive efforts toward the development of dual-emission light-conversion materials [13,14]. For instance, Ding et al. constructed a dual-emission composite by physically embedding blue-emissive carbon dots and Rhodamine B into a metal–organic framework (MOF) matrix [15]. However, physical mixing approaches make it difficult to precisely control the distance and spatial orientation between different emissive components, leading to uncontrollable fluorescence resonance energy transfer (FRET) [16]. Moreover, component migration during prolonged use may induce spectral drift, compromising spectral stability [17]. In view of these limitations, intrinsically dual-emission CQD systems have attracted increasing attention because of their integrated structural advantages [18]. Yang et al. systematically elucidated the FRET-dominated luminescence mechanism in systems featuring the coexistence of carbon-core states and molecular states [19], providing important insight into the origin of multicolor emission in CQDs and establishing a theoretical basis for the rational design of dual-emission systems. Nevertheless, the energy-transfer pathways between emissive centers in such systems still lack effective interfacial structural regulation, rendering the FRET process highly spontaneous and difficult to control. Therefore, further investigation into precise modulation strategies remains highly desirable [20,21,22].

Polyvinyl alcohol (PVA) has been widely employed in light-conversion agricultural films owing to its excellent light transmittance, biodegradability, and abundant modifiable hydroxyl (–OH) functional groups [23,24,25]. For instance, Li et al. fabricated a red-light-conversion agricultural film by blending red-emissive CQDs with PVA, resulting in a 10.4% increase in the fresh weight of bean sprouts [26]. However, the physical blending strategy is prone to CQDs leaching and poor film stability [27]. Moreover, PVA films readily absorb water and undergo swelling, making it difficult to maintain adequate mechanical strength [28,29]. Recent studies have shown that γ-glycidoxypropyltrimethoxysilane (KH-560) contains silane alkoxy groups and epoxy groups [30], which can chemically bond with hydroxyl and amino groups, respectively, thereby effectively improving the mechanical properties [31,32], wettability, swelling behavior, and thermal stability of polymeric materials [33]. For example, Li et al. used KH-560 to crosslink chitosan and prepared hydrogels with low swelling, high mechanical strength, and environmental responsiveness [34].

**Scheme 1:**
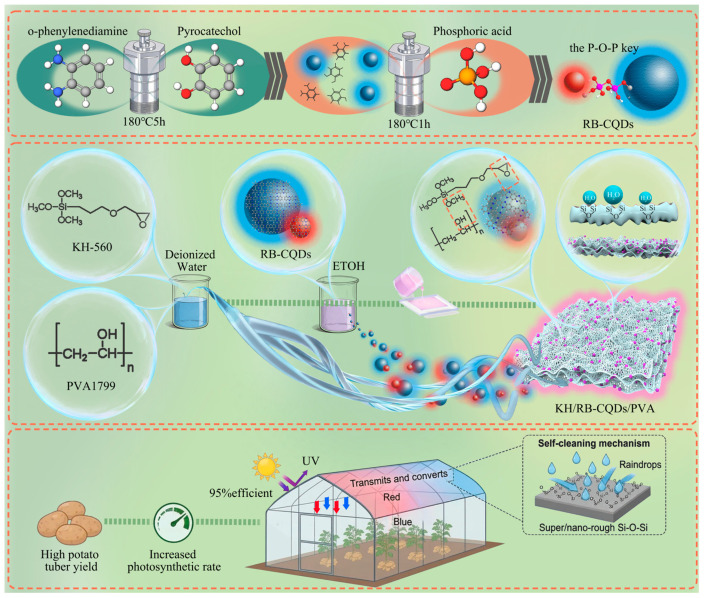
Fabrication of a self-cleaning red–blue dual-emissive light-converting agricultural film as well as the applications of potato.

Herein we report a silane-crosslinked red/blue dual-emission carbon quantum dot/polyvinyl alcohol composite agricultural film (KH/RB-CQDs/PVA) with self-cleaning capability, fabricated through a covalent anchoring strategy. Blue-emissive carbon cores are first generated via hydrothermal treatment, followed by phosphoric acid-assisted secondary treatment that converts residual precursors into red-emissive molecular species covalently bridged through pyrophosphate linkages. This rigid bridging architecture suppresses FRET and spatially separates the two emissive centers, endowing the RB-CQDs with excellent photostability. Subsequently, KH-560 is employed to construct a robust covalent crosslinked network that anchors RB-CQDs within PVA while simultaneously forming a rough Si-O-Si surface structure. The resulting film exhibits superior mechanical strength, high UV shielding efficiency, and outstanding hydrophobic self-cleaning performance (Scheme 1). More importantly, chlorophyll fluorescence kinetics, gas-exchange analyses, and photosynthetic response curves demonstrate that the delivered red/blue composite spectrum synergistically optimizes photosystem II electron transport and RuBisCO-mediated carbon assimilation, leading to significantly enhanced potato net photosynthetic rate and tuber yield. This work provides a molecular design principle for high-performance intelligent agricultural films.

## 2. Experimental Section

### 2.1. Materials

O-phenylenediamine (OPD), catechol (CAT), KH-560 (97%), and poly(vinyl alcohol)-1799 (PVA-1799) were purchased from Macklin (AR, Shanghai, China). H_3_PO_4_ were obtained from Kelong (AR, Chengdu, China). Ultrapure water (resistivity: 18.25 MΩ·cm^−1^) was used in all experiments. All chemicals and solvents were used as received without further purification.

### 2.2. Preparation of CQDs

B-CQDs, G-CQDs, and RB-CQDs were synthesized following a common hydrothermal procedure with specific secondary treatments. Initially, OPD (1.0 g, 9.25 mmol)) and CAT (1.0 g, 9.09 mmol) were dissolved in 50 mL of ultrapure water, sonicated for 20 min, and heated in an 80 mL Teflon-lined stainless-steel autoclave at 180 °C for 5 h. After natural cooling to room temperature, the mixtures were subjected to different sequential steps: the solution for B-CQDs received no further treatment; for G-CQDs, the mixture underwent a secondary hydrothermal treatment at 180 °C for 1 h; and for RB-CQDs, 1250 μL of H_3_PO_4_ was added to the mixture prior to an identical secondary hydrothermal heating (180 °C, 1 h). Finally, all resulting dispersions were cooled, filtered through a 0.22 μm membrane, dialyzed against ultrapure water (MWCO 1000 Da) for 48 h (with water refreshed every 2 h), and freeze-dried to yield the respective CQDs.

### 2.3. Preparation of PVA, RB-CQDs/PVA, and Self-Cleaning KH/RB-CQDs/PVA Films

The PVA, RB-CQDs/PVA, and self-cleaning KH/RB-CQDs/PVA films were prepared using a standardized solution-casting method. Initially, a base PVA solution was prepared by dissolving PVA-1799 (3 g, degree of polymerization ~1700, hydrolysis 99%) is dissolved in 60 mL deionized water at 95 °C for 2 h under reflux. Concurrently, an RB-CQDs dispersion exhibiting red–blue dual emission was prepared by dispersing 5 mg of RB-CQDs powder in 5 mL of ethanol. The specific casting mixtures were then formulated as follows: the base PVA solution was used directly for the pristine PVA film; for the RB-CQDs/PVA film, 1.5 mL of the RB-CQDs (1 mg/mL) solution was added to the base PVA solution and stirred thoroughly; for the self-cleaning KH/RB-CQDs/PVA film, 2 mL of KH-560 (density 1.07 g/mL, 7.6 mmol) and 1.5 mL of the RB-CQDs (1 mg/mL) solution were sequentially added to the base PVA solution and stirred until homogeneous. Finally, each resulting solution was cast into a mold and dried under a dry atmosphere to yield the respective films. Drying conditions are now defined as 40 °C and 50% relative humidity for 48 h.

### 2.4. Characterization

Scanning electron microscopy (SEM, JSM-7500F, JEOL, Japan) and transmission electron microscopy (TEM, JEM-ARM200FTH, JEOL, Japan) were employed to investigate the morphology and crystal structure of the materials, and an energy-dispersive X-ray spectrometer (EDS) was used to record the elemental distributions on the film surfaces.

X-ray diffraction (XRD, DX-2600, China) was used to determine the crystal structure of the synthesized products. Fourier-transform infrared (FTIR) spectroscopy (Nicolet iN10, Thermo Fisher Scientific, USA) and X-ray photoelectron spectroscopy (XPS, XSAM800, UK) were carried out to analyze the chemical composition of the nanocomposites.

Photoluminescence (PL) spectra in the 200–800 nm wavelength range were recorded using a fluorescence spectrometer (FLS1000, UK). A UV–vis–NIR spectrophotometer (UV-3600i Plus, Japan) was used to measure the UV absorbance and visible-light transmittance of the films. An open-frame confocal micro-Raman spectrometer (DeepBlue2000, China) was employed to evaluate the graphitization degree of the carbon structures.

Liquid chromatography–mass spectrometry (LC–MS, Q Exactive Plus, Thermo Fisher Scientific, USA) was used to identify molecular fragments formed during CQD synthesis.

### 2.5. Evaluation of Film Structure and Self-Cleaning Performance

To comprehensively evaluate the multi-functional properties of the PVA, RB-CQDs/PVA, and KH/RB-CQDs/PVA composite films, a series of standardized tests were conducted. Self-cleaning performance was assessed by dropping 2 mL of a blue turbid methylene blue (BTM) suspension (prepared with 0.3 g soil and 50 mL of 1 mg mL^−1^ MB) onto films (2.5 × 4 cm) inclined at a 45° angle, calculating the residual BTM mass gravimetrically. For the dust-proof assay, 0.36 g of dust was evenly spread onto the film surfaces, exposed to airflow from a fan positioned 20 cm away for 20 min, and the dust-removal efficiency was determined via mass difference. Anti-bacterial adhesion was evaluated against Staphylococcus aureus and Escherichia coli using a plate-counting method: 1 × 1 cm film pieces were co-incubated in bacterial suspensions for 24 h, followed by ultrasonic detachment (2 min), serial dilution (10^5^–10^9^), and plating on LB agar (37 °C, 12 h) for colony quantification. The mechanical properties (stress–strain curves) were determined at 25 °C using a universal testing machine (UTM4103X) with film thickness measured via a micrometer, averaging the results from three independent tests per sample. Finally, dynamic water contact angles were measured using the sessile drop method (SDC-350 goniometer) by depositing 2.0 μL of deionized water to monitor the time-dependent hydrophilicity of the surfaces. All measurements were performed with the following replication numbers: fluorescence intensity and lifetime (n = 3 technical replicates per sample), tensile testing (n = 5 specimens per film type), self-cleaning residue rate (n = 3 independent experiments), water contact angle (n = 5 measurements per sample), and antibacterial adhesion (n = 3 independent replicates).

### 2.6. Potato Cultivation and Related Tests

Chuanyu 117 potato seedlings were planted in Ya’an, Sichuan (China) and grown under a simple greenhouse film structure with shading treatment. Three groups were established: PVA, CK, and KH/RB-CQDs/PVA, each with nine biological replicates (independent potato plants). Technical replicates consisted of three repeated measurements per plant, which were averaged before statistical analysis. Three independent planting trials were conducted across two growing seasons; data from all three trials were pooled (total n = 27 per treatment). After 45 days of growth, measurements were taken. Photosynthetic parameters of the potatoes were determined using a LI-COR 6800 portable photosynthesis system (Lincoln, NE, USA). Enzyme activity was measured using a 1,5-ribulose bisphosphate carboxylase (RuBisCO) ELISA assay kit (China). Chlorophyll fluorescence parameters were measured using a MINI-PAM-II chlorophyll fluorometer (Germany). Environmental conditions during potato cultivation were monitored daily: temperature (day 25 ± 2 °C, night 18 ± 2 °C), relative humidity (60 ± 10%), photosynthetically active radiation (PAR, 800 ± 100 μmol·m^−2^·s^−1^ at noon), and soil moisture (maintained at 70 ± 5% field capacity). No significant disease pressure was observed during the trials. Nutrient solution (Hoagland’s) was applied weekly. All groups received identical environmental and nutritional management.

## 3. Results and Discussion

To elucidate the formation mechanism of the RB-CQDs and the critical role of phosphoric acid, a comparative study was conducted among three samples synthesized via distinct pathways: (i) blue-emissive carbon quantum dots (B-CQDs) obtained via one-step hydrothermal treatment of o-phenylenediamine (OPD) and catechol (CAT), serving as the blue-emissive carbon core; (ii) green-emissive carbon quantum dots (G-CQDs) derived from a secondary hydrothermal treatment of the B-CQD precursor without any additives, representing a carbon-core growth pathway; and (iii) the RB-CQDs synthesized by introducing phosphoric acid (H_3_PO_4_) during the secondary hydrothermal step. This systematic comparison allows us to decouple the effects of prolonged reaction time from the specific chemical intervention of phosphate.

**Figure 1:**
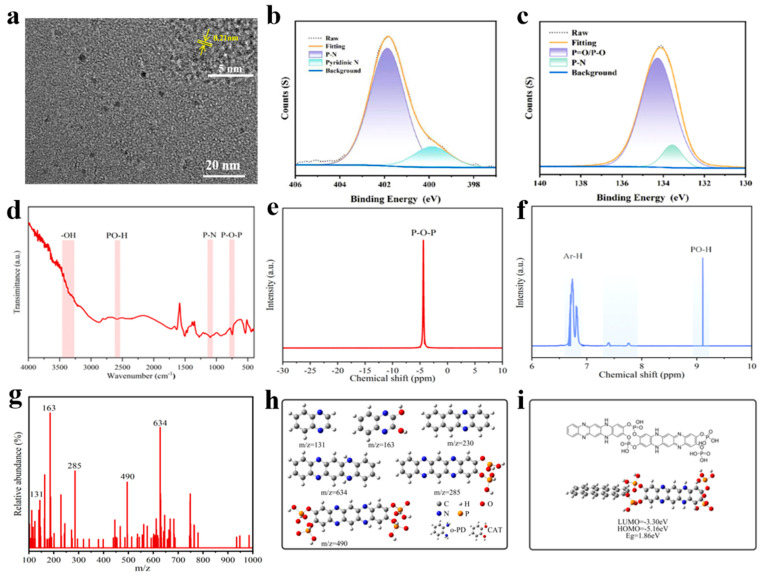
(**a**) TEM image of RB-CQDs (inset: HRTEM image), (**b**) High-resolution N 1s spectrum, (**c**) High-resolution P 2p spectrum, (**d**) FT-IR spectrum, (**e**) ^31^P NMR spectrum, (**f**) ^1^H NMR spectrum, (**g**) Mass spectrum, (**h**) Mass spectral fragmentation analysis, (**i**) DFT-calculated HOMO, LUMO, and Eg of RB-CQDs.

Transmission electron microscopy (TEM) images (Figure 1a and Figure S1) revealed that all three CQDs possessed monodispersed quasi-spherical morphologies without obvious aggregation. High-resolution TEM (HRTEM) showed lattice fringes with a spacing of 0.21 nm, corresponding to the (100) plane of graphitic carbon, confirming the existence of localized sp^2^-conjugated domains (Figure 1a, insets). Crucially, particle size distribution analysis (Figure S2) unveiled a clear structural evolution trajectory: the size increase from B-CQDs (2.11 nm) to G-CQDs (3.02 nm) was consistent with the fluorescence redshift from 441 nm to 529 nm (Figure S3), reflecting a quantum confinement effect [35]. Notably, RB-CQDs exhibited a significantly smaller particle size (2.85 nm) compared to G-CQDs, despite undergoing the same secondary hydrothermal treatment. This size reduction can be attributed to the dual regulatory function of phosphoric acid: (i) coordination-mediated suppression of excessive carbon-core growth, and (ii) surface passivation through pyrophosphate bond formation, which effectively modulates the interface between carbon-core states and molecular states. X-ray diffraction (XRD) patterns (Figure S4) showed that all samples exhibited typical amorphous structural characteristics, and the broad peak centered at 25° reflected short-range ordered sp^2^-conjugated domains, consistent with the HRTEM results [36]. Among them, B-CQDs displayed the weakest diffraction intensity, indicating a shorter effective conjugation length, which may originate from the lower internal ordering of the carbon cores [37]. In contrast, the diffraction peak of RB-CQDs became distinctly narrower and sharper, while additional weak diffraction peaks appeared at 15°, 17°, and 20°, indicating the presence of some ordered structures within the otherwise amorphous carbon matrix; the exact nature of these phases requires further investigation [38]. Combined with Raman spectroscopy analysis (Figure S5), the I_D/I_G ratio of B-CQDs was determined to be 0.91, whereas this value decreased to 0.76 for G-CQDs after secondary hydrothermal treatment, indicating a higher degree of graphitization [39]. This was attributed to the further ordering or growth of sp^2^ domains within the carbon cores during the secondary hydrothermal process [40]. In sharp contrast, the I_D/I_G ratio of RB-CQDs obtained by introducing phosphoric acid during the secondary hydrothermal treatment increased to 0.95, indicating that the overall structural disorder and defect density were significantly enhanced [41]. This phenomenon can be explained by two mechanisms: (i) phosphoric acid suppresses the excessive growth and regular arrangement of carbon cores through coordination effects, and (ii) phosphorus incorporation introduces new structural defects, such as P–N and P–O bonding configurations, thereby enhancing the relative intensity of the D band. These Raman results, together with TEM and XRD observations, collectively indicate that phosphoric acid fundamentally alters the carbonization pathway during the secondary hydrothermal process, promoting the formation of CQDs with higher defect density and abundant interfacial structures. At first glance, a higher I_D/I_G ratio (more disorder) seems inconsistent with the enhanced photostability observed for RB-CQDs. However, the increased disorder originates primarily from edge defects and heteroatom (P, N) incorporation, which create localized energy traps that do not promote photooxidation. Meanwhile, the pyrophosphate bridges formed by phosphoric acid passivate reactive surface sites, preventing oxygen-mediated degradation. Thus, disorder and photostability are not mutually exclusive; the covalent bridges provide a protective “armor” around the carbon core, while the heteroatom-induced defects do not accelerate photobleaching. The X-ray photoelectron spectroscopy (XPS) survey spectrum (Figure S6) showed that RB-CQDs contained C, O, N, and P elements, confirming the successful incorporation of phosphorus. The high-resolution C 1s spectrum of RB-CQDs (Figure S6) exhibited characteristic peaks at 284.8 eV (C=C/C–C), 286.3 eV (C–O/C–N), 286.9 eV (C=N), and 288.7 eV (C=O). The O 1s spectrum (Figure S6) could be deconvoluted into three peaks assigned to C=O (531.3 eV), C–O/–OH (532.5 eV), and P–O (532.9 eV), respectively. In the N 1s spectrum (Figure 1b), the peaks at 399.8 eV (pyridinic N) and 401.8 eV (graphitic N) confirmed the doping state of nitrogen, while the P–N signal at 401.9 eV further revealed the chemical bonding between phosphorus and the carbon cores. In the P 2p spectrum (Figure 1c), the peaks at 133.5 eV (P–N) and 134.1 eV (P=O) further verified that phosphorus existed on the surface of RB-CQDs in multiple bonding forms. Together with the Fourier transform infrared (FT-IR) spectrum (Figure 1d), in which the characteristic peaks at 752 cm^−1^ (P–O–P vibration), 1086 cm^−1^ (P–N vibration), and 2560 cm^−1^ (P–O–H) were observed, these results directly confirmed that phosphorus was bonded in the forms of P=O, P–O, and P–N [42]. Notably, the –OH peak at 3450 cm^−1^ in RB-CQDs was significantly reduced compared with those of B-CQDs and G-CQDs (Figure S7). In addition, the ^31^P nuclear magnetic resonance (^31^P NMR) spectrum (Figure 1e) showed a characteristic signal of pyrophosphate bonds (P–O–P), indicating that phosphoric acid replaced hydroxyl groups to form pyrophosphate linkages bridging the carbon cores and surface molecular moieties [43]. In this way, phosphoric acid acted as a “molecular bridge” and ultimately constructed RB-CQDs with a multifunctional integrated structure. To identify the molecular fluorophores responsible for red emission, ^1^H nuclear magnetic resonance (^1^H NMR) spectroscopy was performed (Figure 1f). RB-CQDs exhibited two types of aromatic proton signals in the range of 6.6–7.9 ppm. Signals at 6.6–6.9 ppm were assigned to aromatic protons, whereas those at 6.9–7.9 ppm were consistent with the protonation characteristics of the fluorophore 5,14-dihydroquinoxalino [2,3-b]phenazine (DHQP) [22]. Notably, a sharp singlet appeared at 9.1 ppm in RB-CQDs, assigned to the proton resonance of surface P–OH groups, confirming that phosphoric acid participated in structural modification through hydroxyl condensation. High-performance liquid chromatography–mass spectrometry (HPLC-MS) analysis (Figure 1g) further confirmed these findings, with the peak at *m*/*z* = 285 corresponding to the [M + H]^+^ ion of DHQP [22], verifying the presence of covalently connected fluorescent molecular moieties on the carbon-core surface. Fragment analysis (Figure 1h) showed that peaks at *m*/*z* = 133, 163, and 230 may originate from polycyclic aromatic or heterocyclic structural fragments derived from OPD and CAT, whereas peaks at *m*/*z* = 490 and 634 may correspond to different linkage forms between DHQP and pyrophosphate bonds. The assignment of DHQP as the red-emitting molecular fluorophore is supported by the excellent agreement between our HPLC-MS, ^1^H NMR, and fragment analysis data and those reported by Li et al. for the same OPD-catechol system. Density functional theory (DFT) calculations were performed to further explore the formation mechanism and electronic structure of RB-CQDs (Figure 1i). Based on the B3LYP/6-31G(d,p) basis set, a simulated geometric model was constructed using the small molecules identified by mass spectrometry. The structural simulation indicated that during the phosphoric acid-assisted secondary hydrothermal process, DHQP was bridged with the sp^2^ structure through pyrophosphate bonds to form RB-CQDs, with a calculated bandgap energy (E_g) of 1.86 eV. According to Tauc plot results (Figure S8), the experimental bandgap energy of RB-CQDs was 1.75 eV, closely matching the simulated value [44]. The consistency between theoretical calculations and experimental characterization not only verified the rationality of the structural model in which pyrophosphate bonds bridge carbon cores and molecular fluorophores but also provided atomic-scale evidence supporting the feasibility of the carbon-core/molecular-state regulation strategy. Collectively, these structural insights—specifically the pyrophosphate-bridged architecture and the identified DHQP fluorophores—provide a solid foundation for the observed dual-emission behavior. The photoluminescence spectra (Figure S3) preliminarily confirm this design, showing that RB-CQDs exhibit distinct dual emission peaks at 462 and 625 nm under 365 nm excitation, in sharp contrast to the single-mode emissions of B-CQDs (441 nm) and G-CQDs (529 nm). Building on this structure–optical correlation, we will next dissect the underlying photophysical mechanisms and microenvironment-dependent emission dynamics.

**Figure 2:**
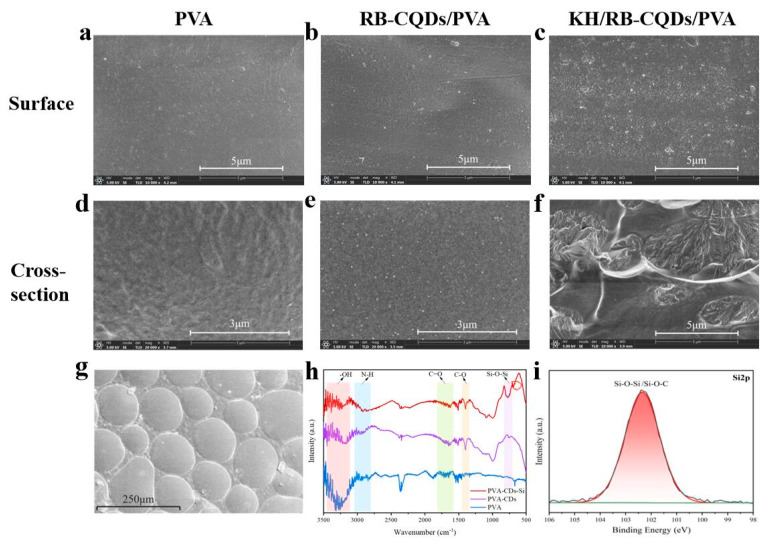
(**a**–**f**) SEM images of the surface and cross-section of PVA, RB-CQDs/PVA, and KH/RB-CQDs/PVA films, (**g**) EDS analysis of KH/RB-CQDs/PVA, (**h**) FT-IR spectra of PVA, RB-CQDs/PVA, and KH/RB-CQDs/PVA films, (**i**) high-resolution Si 2p XPS spectrum of the KH/RB-CQDs/PVA film.

To achieve effective immobilization of RB-CQDs within the polymer matrix and construct a hydrophobic interface, the silane coupling agent KH-560 was employed through a molecular bridging strategy. To further investigate the microstructure of the films, the surface and cross-sectional morphologies were characterized by scanning electron microscopy (SEM). As shown in (Figure 2a–f), the surface of the pure PVA film was smooth, whereas both the RB-CQD/PVA and KH/RB-CQD/PVA films exhibited a certain degree of roughness. The increased surface roughness of the RB-CQDs/PVA film mainly originated from the embedding and distribution of CQD nanoparticles within the PVA matrix. These nanoscale CQDs acted as physical topological sites, preliminarily establishing the basis for a micro/nanoscale hierarchical roughness, thereby enhancing the hydrophobicity of the matrix [45]. In contrast, the surface roughness of the KH/RB-CQD/PVA film increased much more significantly, which was mainly attributed to the multistep covalent reactions triggered by KH-560 and their synergistic effects. This process constructed a robust internal covalent network through: (i) Si–O–C bond formation between silane alkoxy groups and PVA hydroxyl groups, and (ii) ring-opening bonding between epoxy groups and surface functional groups of RB-CQDs [31,32]. More importantly, residual silane underwent hydrolysis and condensation on the film surface, thereby in situ generating a dense micro/nanoscale hierarchical Si–O–Si rough structure [46]. As a result, surface energy was markedly reduced, endowing the film with stable and excellent hydrophobic performance. Energy-dispersive X-ray spectroscopy (EDS) elemental mapping images (Figure 2g and Figure S9) further confirmed that Si (from KH-560), C, O, N, and P (from RB-CQDs) were uniformly distributed throughout the PVA matrix, indicating that RB-CQDs were homogeneously immobilized through the coupling effect of KH-560 and that a biomimetic rough surface structure was successfully formed. Fourier transform infrared (FT-IR) spectra (Figure 2h) showed that the broad peak at 3350 cm^−1^ was assigned to the stretching vibration of –OH. Compared with pure PVA, both RB-CQDs/PVA and KH/RB-CQDs/PVA exhibited characteristic absorption peaks at 2980 cm^−1^ (N–H), 1750 cm^−1^ (C=O), and 1350 cm^−1^ (C–O), confirming the successful incorporation of RB-CQDs. In particular, KH/RB-CQDs/PVA displayed a characteristic vibrational peak of Si–O–Si bonds at 750 cm^−1^, indicating that KH-560 had successfully participated in the reaction and formed a siloxane crosslinked network, which further supported the formation mechanism of the hydrophobic interface [47]. The X-ray photoelectron spectroscopy (XPS) survey spectrum (Figure S10) showed that the composite consisted of C, O, N, P, and Si, confirming the successful introduction of KH-560 and RB-CQDs. Further high-resolution spectral analysis revealed that the fine chemical states of the elements in the composite were highly consistent with the characteristics of RB-CQDs. Specifically, the peaks in the C 1s spectrum at 284.8, 286.3, 286.9, and 288.7 eV were assigned to C=C/C–C, C–O/C–N, C=N, and C=O bonds, respectively. The O 1s spectrum could be deconvoluted into three components at 531.3 eV (C=O), 532.5 eV (C–O/–OH), and 532.9 eV (P–O). In the N 1s spectrum, the peaks at 399.8 and 401.8 eV corresponded to pyridinic N and graphitic N, respectively, while the P–N signal at 401.9 eV further verified the bonding interaction between P and the carbon cores. In the P 2p spectrum, the peaks at 133.5 eV (P–N) and 134.1 eV (P=O) indicated that phosphorus existed on the material surface in multiple chemical states. These results collectively demonstrated that RB-CQDs had been successfully incorporated into the composite while retaining their original chemical structure. In addition, the Si 2p spectrum (Figure 2i) showed a distinct Si–O–Si/Si–O–C characteristic peak at 102.07 eV, which further confirmed that KH-560 had been successfully grafted onto the PVA matrix through hydrolysis and condensation. More importantly, this covalent network constructed a rigid microenvironment that would suppress nonradiative transitions of the molecular-state fluorophores, as will be demonstrated in the optical performance analysis.

**Figure 3:**
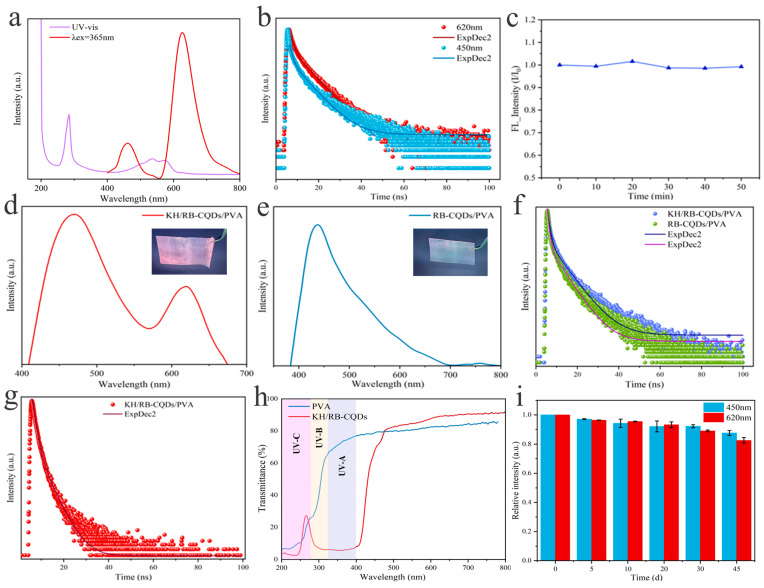
(**a**) UV-vis absorption, excitation, and emission spectra of RB-CQDs, (**b**) Fluorescence decay curves of RB-CQDs, (**c**) Fluorescence intensity of RB-CQDs after 50 min of continuous UV irradiation, (**d**) Fluorescence spectra of KH/RB-CQDs/PVA under 365 nm excitation, (**e**) Fluorescence spectra of RB-CQDs/PVA under 365 nm excitation, (**f**) Fluorescence decay curves of KH/RB-CQDs/PVA and RB-CQDs/PVA at 450 nm, (**g**) Fluorescence decay curve of KH/RB-CQDs/PVA at 620 nm, (**h**) UV-vis-NIR spectra of KH/RB-CQDs/PVA and PVA, (**i**) Long-term fluorescence stability of KH/RB-CQDs/PVA.

The ultraviolet–visible absorption and photoluminescence spectra (Figure 3a) revealed the unique optical characteristics of RB-CQDs. Characteristic absorption peaks appeared at 280 nm, attributed to the π–π* transition of C=C/C=N bonds in the carbon cores [48], and at 400 nm, originating from the n–π* transition of surface C–N/C–O groups [49]. Notably, pronounced molecular-state absorption bands at 540 and 580 nm arose from fluorescent molecular clusters anchored onto the carbon cores through pyrophosphate linkages. This unique structure enabled RB-CQDs to exhibit dual-mode photoluminescence under 365 nm excitation, including blue emission at 462 nm and red emission at 625 nm. The blue emission originates from the carbon core (like a tiny piece of graphite), while the red emission comes from small dye-like molecules (DHQP) attached to the core’s surface. The pyrophosphate bonds act as ‘rigid ropes’ that hold the dye molecules at a fixed distance from the core, preventing energy transfer between them. This spatial separation ensures both colors are stable and bright. The dual-emission behavior was further confirmed by time-correlated single-photon counting (TCSPC) measurements. As shown in (Figure 3b, Figures S11 and S12), the decay traces of RB-CQDs under 365 nm excitation displayed a multi-exponential decay behavior, indicating the presence of two emissive centers in RB-CQDs. The shorter lifetime was attributed to molecular-state fluorescence on the carbon-core surface, whereas the longer lifetime originated from carbon-core-state fluorescence [22]. These results indicated that the red fluorescence from the molecular states and the blue fluorescence from the carbon-core states acted synergistically, giving rise to the unique red/blue dual-emission characteristic. In addition, the optical stability of RB-CQDs was systematically evaluated. The stable structure constructed by pyrophosphate-bridging enabled the formation of an electronically coupled system between the carbon cores and the surface molecular states via covalent bonds, which facilitates radiative charge recombination, while the spatial separation simultaneously suppresses non-radiative FRET. This dual mechanism thereby significantly improving the overall stability of the material [50]. As shown in (Figure 3c and Figure S13), the dual-emission fluorescence intensity of RB-CQDs remained essentially unchanged after 50 min of continuous ultraviolet (UV) irradiation and 30 d of storage, demonstrating excellent photostability. It is important to note that electronic coupling (through bonds) and FRET suppression (through spatial isolation) are not contradictory; they operate via different physical mechanisms and together enhance photostability.

Having established the covalent anchoring configuration, we systematically compared the photophysical properties of KH/RB-CQDs/PVA and RB-CQDs/PVA to elucidate how this rigid network governs the optical behavior of RB-CQDs and the resulting light-conversion mechanism in the composite films. Under 365 nm UV excitation, the KH/RB-CQDs/PVA film exhibited characteristic emission peaks at 450 and 620 nm that were consistent with those of the RB-CQDs solution (Figure 3d), whereas RB-CQDs/PVA showed only the 450 nm emission, and the red emission at 620 nm was completely quenched (Figure 3e). This striking contrast confirmed that the red emission originated from molecular-state fluorophores that were highly sensitive to the microenvironment [51]. In the physically blended film, the high density of hydroxyl groups in the PVA matrix formed a strong hydrogen-bonding network with the emissive molecular states, providing nonradiative decay pathways for the excited-state energy and thereby selectively quenching the red emission [52]. In contrast, KH-560 constructed a covalent crosslinked network between RB-CQDs and the PVA matrix, which effectively suppressed the nonradiative transitions of the surface states and restricted excited-state energy dissipation [53]. As a result, the emissive process was stabilized, the surface-quenching effect was eliminated, and the retention of dual-emission behavior together with the significant enhancement of fluorescence performance was achieved. These results highlight the critical role of the covalent crosslinking strategy in constructing high-performance solid-state fluorescent composites. The fluorescence lifetime results further revealed the optimization effect of covalent bonding on the emission dynamics. Compared with the solution state (450 nm: 2.56 ns; 620 nm: 2.79 ns), the lifetimes of the carbon dots in the composite film were significantly prolonged in both emission channels (450 nm: 7.12 ns; 620 nm: 9.25 ns) (Figure 3f,g, Figures S14 and S15). This phenomenon indicated that the covalent crosslinked network constructed by the silane coupling agent effectively passivated the defect sites in the carbon-core states and surface molecular states, thereby suppressing nonradiative recombination pathways [54]. Notably, the control sample without KH-560 crosslinking showed only a limited increase in lifetime (Figure 3f and Figure S16), further confirming the key role of covalent anchoring in enhancing interfacial compatibility and stabilizing the emissive centers. Further quantum yield measurements supported this conclusion from an energetic perspective: the fluorescence quantum yield increased markedly from 1.14% in solution to 16.16% in the film (Figures S17 and S18), indicating that the covalent network optimized the radiative recombination process of excitons [55]. Although 16.16% is moderate compared to green or blue emissive CQDs, it is notably high for red-emissive CQDs, which typically exhibit solution-phase QYs below 10%. The ultraviolet–visible–near-infrared spectra showed (Figure 3h) that the film could block approximately 95% of UV radiation in the range of 200–400 nm, while maintaining a transmittance of over 80% in the visible region, thereby achieving effective shielding of harmful UV light together with high visible-light transmittance. In addition, after 45 d of use (Figure 3i), the film still retained excellent red/blue fluorescence intensity at over 85%, demonstrating good photostability and satisfying the durability requirements for the practical application of light-conversion agricultural films.

**Figure 4:**
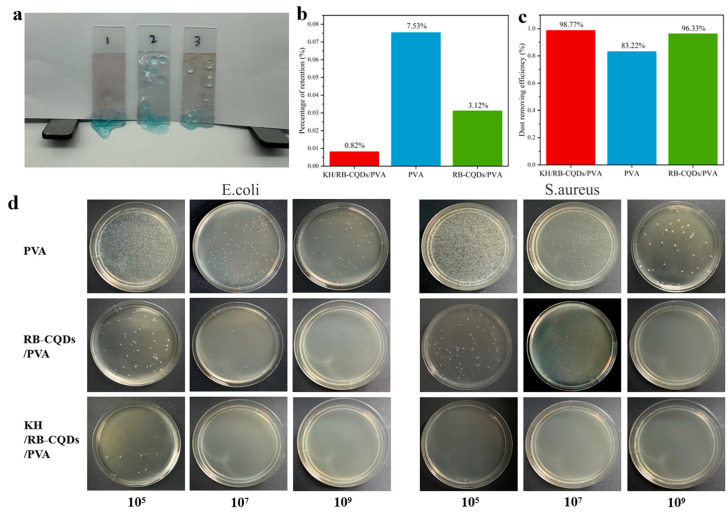
Surface performance comparison of PVA, RB-CQDs/PVA, and KH/RB-CQDs/PVA films: (**a**) Surface pollutant retention images, (**b**) pollutant residue ratios, (**c**) dust-removal efficiencies, (**d**) antibacterial adhesion performance.

The water contact angles (WCAs) of PVA, RB-CQDs/PVA, and KH/RB-CQDs/PVA were measured using an automatic contact angle meter. As shown in (Figure S19), the WCA of PVA was 43.9°, whereas those of RB-CQDs/PVA and KH/RB-CQDs/PVA increased to 95.58° and 111.6°, respectively. These results demonstrated that KH/RB-CQDs/PVA possessed excellent hydrophobicity, highlighting its strong potential as a self-Cleaning light-conversion agricultural film. The self-cleaning performance was further evaluated using a blue turbid methylene blue (BTM) solution. As shown in (Figure 4a), almost no BTM residue was observed on the surface of KH/RB-CQDs/PVA, indicating a remarkable self-cleaning effect. Quantitative analysis of the residue rate (Figure 4b) showed that the residue rates of PVA, RB-CQDs/PVA, and KH/RB-CQDs/PVA were 7.53%, 3.12%, and 0.82%, respectively, further confirming that KH/RB-CQDs/PVA exhibited the best self-cleaning performance. The dustproof performance of the films was also evaluated under simulated natural wind conditions, as shown in (Figure S20 and Figure 4c). With increasing exposure time, KH/RB-CQDs/PVA exhibited the highest dust removal efficiency of 98.77%, which was significantly superior to that of PVA (83.22%) and RB-CQDs/PVA (96.33%). This outstanding dustproof property was likely related to its lower surface energy, indicating promising practical value in agricultural covering applications [56]. In addition, the anti-adhesion performance of the films against Escherichia coli and Staphylococcus aureus was investigated using the plate counting method. As shown in (Figure 4d), a large number of bacterial colonies were still observed on the PVA surface after 10^5^-fold dilution of the bacterial suspension, whereas almost no colonies formed on RB-CQDs/PVA and KH/RB-CQDs/PVA. In particular, even at a higher dilution ratio of 10^9^, KH/RB-CQDs/PVA still exhibited the best anti-adhesion performance against bacteria. This anti-adhesion behavior could mainly be attributed to the multiscale micro/nanostructure, which prevented direct bacterial contact, together with the low surface energy, which further suppressed bacterial adhesion [57]. Morphological stability tests showed that after immersion in water for 30 min, KH/RB-CQDs/PVA did not undergo noticeable deformation (Figure S21), whereas PVA and RB-CQDs/PVA exhibited warping and wrinkling, indicating the superior moisture resistance of KH/RB-CQDs/PVA. Tensile tests performed using a universal testing machine (Figure S22) clearly revealed the mechanical properties of the films and their environmental stability. Under normal conditions, the incorporation of RB-CQDs significantly increased the tensile strength and elongation at break of PVA by 98.41% and 124.69%, respectively. After the further introduction of KH-560 to construct a covalent crosslinked network, these values were markedly increased by 291.05% and 160.12%, respectively. Although PVA itself possesses excellent film-forming ability, its mechanical properties are easily lost in practical agricultural applications because of water absorption and swelling. To evaluate the water resistance of the materials, the stress–strain behaviors of the three films after immersion treatment were further measured. The results showed that the mechanical properties of pure PVA sharply deteriorated after water exposure. Although the mechanical performance of RB-CQDs/PVA also decreased, it still remained superior to that of pristine PVA under normal conditions. In contrast, the mechanical properties of KH/RB-CQDs/PVA showed no significant change after immersion, demonstrating excellent structural integrity and environmental durability.

**Figure 5:**
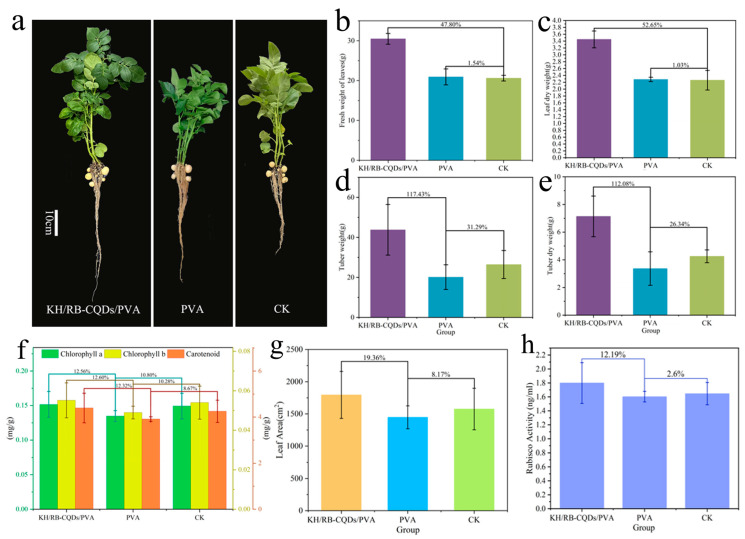
Comparison of potato plants treated with PVA, CK (uncovered control), RB-CQDs/PVA, and KH/RB-CQDs/PVA after 45 days of growth. (**a**) Cultivation images. (**b**) Fresh leaf weight (g/plant, mean ± SD, n = 27). (**c**) Dry leaf weight (g/plant). (**d**) Fresh tuber weight (g/plant). (**e**) Dry tuber weight (g/plant). (**f**) Chlorophyll a, chlorophyll b, and carotenoid contents (mg/g fresh weight). (**g**) Leaf area (cm^2^). (**h**) RuBisCO carboxylase activity (ng/mL, fresh weight). Statistical analysis: one-way ANOVA with Tukey’s HSD post hoc test. Compared to the PVA group, the KH/RB-CQD/PVA group showed significantly higher values for all parameters: fresh and dry leaf weight (*p* < 0.01), fresh and dry tuber weight (*p* < 0.001), chlorophyll a/b and carotenoids (*p* < 0.05), leaf area (*p* < 0.01), and RuBisCO activity (*p* < 0.05). Compared to the CK group, only fresh tuber weight showed a significant increase (*p* < 0.05); other parameters did not differ significantly (*p* > 0.05).

To assess the application potential of the KH/RB-CQD/PVA composite film in agricultural settings, we systematically investigated its effects on potato growth under simulated shading within a natural solar-light environment. After 45 days of cultivation, the KH/RB-CQD/PVA treatment group exhibited significant advantages (Figure 5a). The fresh and dry weights of leaves increased by 47.80% and 52.65%, respectively, compared with the CK group (Figure 5b,c). Meanwhile, the fresh and dry weights of tuber yield were increased by 76% and 105%, respectively, relative to the PVA group (Figure 5d,e). Other morphological indicators were also significantly improved (Figure S23). Specifically, the fresh and dry weights of stems increased by 76.91% and 103.88%, respectively, compared with the CK group, while the fresh and dry weights of roots increased by 26.26% and 29.43%. In addition, the plant height increased by 18.51% compared with the PVA group, which was nearly identical to that of the blank group, whereas the root length increased by 29.78% relative to the blank group. Notably, although the PVA treatment group exhibited phenotypic traits comparable to or even slightly better than those of the blank group in several growth indicators, its final yield remained relatively low. This phenomenon further supports the survival strategy of plants competing for light resources under shading conditions through shade avoidance. In such environments, more photosynthetic assimilates are allocated to the growth of stems, leaves, and roots rather than to tuber accumulation, ultimately resulting in a reduction in economic yield [58]. This hypothesis was further verified by photosynthetic physiological parameters. The analysis of photosynthetic pigment contents showed (Figure 5f) that the contents of chlorophyll a, chlorophyll b, and carotenoids in the KH/RB-CQD/PVA group were significantly higher than those in the PVA group, with increases of 12.56%, 12.60%, and 12.32%, respectively. Compared with the PVA group, the CK group also showed increases of 10.80%, 10.28%, and 8.67%, respectively. Meanwhile, the leaf area of the RB-CQD/PVA group increased by 19.63% compared with that of the PVA group, whereas the CK group showed an increase of 8.67% relative to the PVA group, indicating enhanced photosynthetic assimilation capacity (Figure 5g). In terms of the activity of key enzymes involved in carbon assimilation, the RuBisCO carboxylase activity in the KH/RB-CQD/PVA group was significantly enhanced by 12.19% compared with the PVA control group, indicating that the flux of the Calvin cycle was effectively promoted, thereby providing sufficient carbon sources for biomass accumulation (Figure 5h). Overall, under simulated intercropping shading conditions, the KH/RB-CQD/PVA light-conversion film effectively alleviated low-light stress by enhancing photosynthetic pigment synthesis, improving light energy capture efficiency, and promoting key enzyme activities. Through the synergistic optimization of the photosynthetic process, the film significantly promoted potato growth and yield formation, demonstrating promising application prospects for improving crop light environments in agricultural systems.

**Figure 6:**
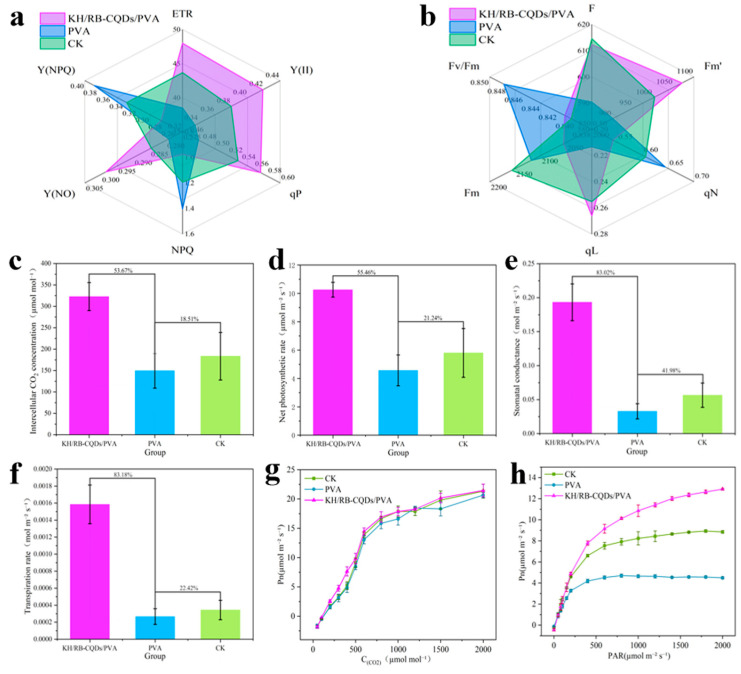
Comparison of photosynthetic parameters of potato plants treated with PVA, CK, RB-CQDs/PVA, and KH/RB-CQDs/PVA. (**a**,**b**) Chlorophyll fluorescence kinetic parameters: Y(II) (actual photochemical quantum yield), ETR (electron transport rate, μmol·m^−2^·s^−1^), qL (fraction of open reaction centers), Y(NO) (non-regulated energy dissipation yield). (**c**) Intercellular CO_2_ concentration (Ci, μmol·mol^−1^). (**d**) Net photosynthetic rate (Pn, μmol CO_2_·m^−2^·s^−1^). (**e**) Stomatal conductance (Gs, mol H_2_O·m^−2^·s^−1^). (**f**) Transpiration rate (Tr, mmol H_2_O·m^−2^·s^−1^). (**g**) Photosynthetic light-response curve (Pn vs. photosynthetically active radiation, PAR, μmol·m^−2^·s^−1^). (**h**) Photosynthetic CO_2_-response curve (Pn vs. Ci). Data are mean ± SD (n = 27). Statistical significance (one-way ANOVA with Tukey’s HSD): For KH/RB-CQDs/PVA vs. PVA, all parameters were significantly improved (*p* < 0.01 for Y(II), ETR, Ci, Gs, Tr; *p* < 0.05 for qL and Y(NO); *p* < 0.001 for Pn). For KH/RB-CQDs/PVA vs. CK, Pn, Gs, Tr, and Ci showed *p* < 0.05; fluorescence parameters were not significantly different (*p* > 0.05).

To systematically elucidate the physiological mechanism by which the KH/RB-CQD/PVA light-conversion film enhances potato yield, a comprehensive analysis was conducted by integrating chlorophyll fluorescence kinetics, gas-exchange parameters, and photosynthetic response curves. The results showed (Figure 6a,b, Figures S24 and S25) that the photosystem II performance of the light-conversion film treatment group was significantly improved. Specifically, the actual photochemical quantum yield (Y(II)), electron transport rate (ETR), and the fraction of open reaction centers (qL) were all the highest, whereas the non-regulated energy dissipation yield (Y(NO)) was the lowest, indicating that light capture and conversion efficiency were comprehensively enhanced while the risk of photodamage was effectively reduced. The gas-exchange parameters (Figure 6c–f) further confirmed this advantage. Compared with the pure PVA group, the treatment group exhibited significant increases of 55.46%, 82.02%, 83.18%, and 53.67% in net photosynthetic rate (Pn), stomatal conductance (Gs), transpiration rate (Tr), and intercellular CO_2_ concentration (Ci), respectively, demonstrating that both leaf carbon assimilation and gas-exchange capacity were substantially enhanced. Notably, all gas-exchange parameters in the blank control group (CK) were significantly higher than those in the pure PVA group, with increases ranging from 20% to 40%. This provides direct evidence for the “etiolation phenomenon”: although pure PVA covering may promote morphological elongation, it actually suppresses stomatal function and carbon assimilation efficiency, thereby forcing the plants into a survival strategy characterized by high consumption but low output.

**Figure 7:**
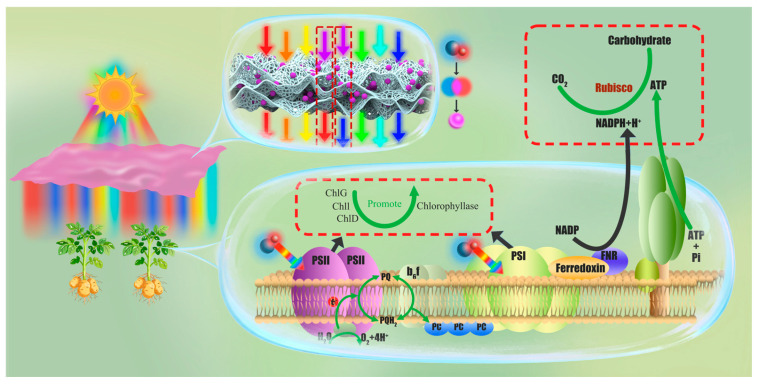
Schematic illustration of enhanced photosynthesis in potatoes induced by KH/RB-CQDs/PVA.

The photosynthetic response curves (Figure 6g) further revealed the intrinsic mechanism. The light-response curve showed that, under different levels of photosynthetically active radiation (PAR), the Pn of the treatment group remained consistently higher than those of the CK and PVA groups, indicating a clear advantage in light-use efficiency across the entire irradiance range. The CO_2_-response curve (Figure 6h) further demonstrated that, under low CO_2_ concentrations, the Pn of the treatment group was significantly higher than that of the control groups, directly confirming a higher carboxylation efficiency of the key carbon-assimilation enzyme RuBisCO. When the CO_2_ concentration increased to the saturation level, the Pn values of the three groups gradually became comparable, indicating that the photosynthetic rate was then mainly limited by the light-reaction capacity. Even under these conditions, the treatment group still exhibited the highest value, which constitutes a complete line of evidence together with the above-mentioned superior photochemical performance, particularly the enhanced Y(II) and ETR. Taken together, these multidimensional results demonstrate that KH/RB-CQD/PVA light-conversion film significantly improves crop photosynthetic efficiency through a three-level synergistic mechanism (Figure 7): (i) promoting synthesis of photosynthetic pigments such as chlorophyll and carotenoids, thereby enhancing light-harvesting capacity; (ii) optimizing photosystem II performance, increasing electron transport rate, and reducing photodamage risk; (iii) activating the activity of RuBisCO, a key carbon-assimilation enzyme, thereby promoting the net photosynthetic rate and enables efficient conversion from light-energy absorption to organic matter synthesis.

To contextualize the performance of our KH/RB-CQD/PVA film within recent literature, we compared it with five representative studies on carbon dot-based agricultural light-conversion films (2021–2025); the detailed comparison is provided in Table S1. Yu et al. [59] developed blue-red dual-emission CQD/PVA films and reported a 152.73% fresh weight increase in bok choy, a leafy vegetable. Although this percentage exceeds our 76% tuber yield increase in potato, the comparison across different crop types (leafy vs. tuber) is not directly equivalent. Notably, our film also enhanced potato shoot fresh weight by 47.8% and dry weight by 52.7%, indicating strong vegetative growth promotion. Moreover, our film uniquely integrates 95% UV shielding, hydrophobic self-cleaning (contact angle 111.6°), and a solid-state quantum yield of 16.16%—properties not quantified or absent in Yu et al.’s work. Ge et al. [60] used biomass-derived CQDs as direct light-conversion materials applied to plants or cyanobacteria, achieving 180% biomass increase in Arabidopsis. However, their approach did not involve a self-standing covering film, which limits direct applicability to greenhouse coverings. Wu et al. [61] fabricated cellulose/CQDs composite films but did not conduct any plant cultivation experiments, leaving the actual agricultural efficacy unvalidated. Li et al. [26] and Li et al. [62] reported yield increases of only 10.4% (mung bean sprouts with red CQDs/PVA) and 19.81% (tomato with foliar-sprayed CDs), both substantially lower than our 76% potato tuber increase. Furthermore, those studies used single-color (red) emission, whereas our red/blue dual-emission design better matches the photosynthetic action spectrum. In summary, while individual previous works may excel in specific metrics (e.g., maximum yield percentage in leafy vegetables), our KH/RB-CQD/PVA film offers the most balanced and multifunctional performance—combining high solid-state QY, UV shielding, self-cleaning, covalent stability, and significant yield enhancement under shading stress—demonstrating the advantage of our covalent anchoring and dual-emission strategy for practical greenhouse applications.

## 4. Limitations and Future Perspectives

Several limitations of the present study should be acknowledged. First, direct measurement of the spectral distribution under the films (red/blue photon flux ratio, PPFD, DLI) was not performed; the proposed light-regulation mechanism is therefore supported only by physiological data. Future work will include such spectral characterization. Second, the greenhouse potato trial was conducted in only one planting season with nine biological replicates per treatment; multi-season field trials are needed to confirm reproducibility under varying environmental conditions. Third, long-term outdoor self-cleaning performance (e.g., under natural rain, wind, and dust accumulation) remains unevaluated; the current laboratory tests provide initial evidence but cannot replace real-weather assessment. Fourth, the synthesis of RB-CQDs involves a two-step hydrothermal process with phosphoric acid, which may increase production costs; continuous-flow synthesis and life-cycle assessment are being explored for scalability. Fifth, while the 76% yield increase under shading treatment is significant, the absolute yield values and shading intensity have been provided to avoid overinterpretation; direct experimental validation of the UV-conversion mechanism (e.g., using UV-blocking filters) was not performed due to the long growth cycle of potatoes and will be addressed in future studies.

## 5. Conclusions

In this study, we successfully developed a weather-resistant light-converting agricultural film based on RB-CQDs using a “structural-zoning” design strategy. The core innovation lies in employing pyrophosphate bridges to covalently couple the carbon core with molecular fluorophores, fundamentally eliminating the spectral instability that typically hinders dual-emissive systems. Furthermore, the KH-560-induced “PVA–silane–CQDs” multilevel crosslinked network rigidly anchors the emissive centers at the molecular scale, suppresses nonradiative transitions, and simultaneously bestows the material with excellent macroscopic mechanical toughness, hydrophobicity, and anti-swelling capability. The resulting KH/RB-CQD/PVA film integrates precise spectral adaptation, efficient UV-to-visible light conversion, self-cleaning behavior, and long-term environmental stability, thereby overcoming the inherent limitations of conventional PVA films. Plant physiological analyses revealed that the KH/RB-CQD/PVA film markedly enhances photosynthetic efficiency through a three-tier synergistic mechanism: (i) promoting the biosynthesis of chlorophylls and carotenoids to enhance light harvesting; (ii) improving PSII performance by increasing electron transport rate and reducing photodamage risk; and (iii) activating RuBisCO carboxylase activity to elevate net photosynthetic rate, thereby enabling more efficient conversion of absorbed light energy into organic carbon. Together, these effects form a coherent mechanistic pathway from light capture to carbon assimilation, ultimately driving significant improvements in potato growth and yield.

## Data Availability

The original contributions presented in this study are included in the article/Supplementary Material. Further inquiries can be directed to the corresponding authors.

## Supplementary Materials

The following supporting information can be downloaded at https://www.mdpi.com/article/10.3390/polym18121442/s1, Figure S1: TEM image of (a) B-CQDs and (b) G-CQDs; Figure S2: Size distribution histogram of (a) B-CQDs (b) G-CQDs and (c) RB-CQDs; Figure S3: Fluorescence emission spectrum of (a) B-CQDs (b) G-CQDs and (c) RB-CQDs; Figure S4: XRD images of B-CQDs, G-CQDs, and RB-CQDs; Figure S5: Raman images of B-CQDs, G-CQDs, and RB-CQDs; Figure S6: XPS spectra of RB-CQDs: (a) survey spectrum, (b) C 1s spectrum, (c) O 1s spectrum; Figure S7: Infrared images of B-CQDs, G-CQDs, and RB-CQDs; Figure S8: Bandgap energy diagram of RB-CQDs; Figure S9: EDS elemental spectra of KH/RB-CQDs/PVA nanocomposite: (a) O spectrum, (b) P spectrum, (c) N spectrum, (d) Si spectrum, (e) C spectrum; Figure S10: XPS spectra of KH/RB-CQDs/PVA nanocomposite: (a) survey spectrum, (b) C 1s spectrum, (c) N 1s spectrum, (d) O 1s spectrum, (e) P 2p spectrum; Figure S11: Fluorescence lifetime of RB-CQDs at 450 nm; Figure S12: Fluorescence lifetime of RB-CQDs at 620 nm; Figure S13: Long-term fluorescence stability of RB-CQDs; Figure S14: Fluorescence lifetime of KH/RB-CQDs/PVA at 450 nm; Figure S15: Fluorescence lifetime of KH/RB-CQDs/PVA at 620 nm; Figure S16: Fluorescence lifetime of RB-CQDs/PVA at 450 nm; Figure S17: Fluorescence quantum yield of RB-CQDs; Figure S18: Fluorescence quantum yield of KH/RB-CQDs/PVA; Figure S19: Water contact angles of PVA, RB-CQDs/PVA, and KH/RB-CQDs/PVA; Figure S20: Dust removal images of PVA, RB-CQDs/PVA, and KH/RB-CQDs/PVA; Figure S21: (a–c) Images before immersion of PVA, RB-CQDs/PVA, and KH/RB-CQDs/PVA, (d–f) Images after immersion of PVA, RB-CQDs/PVA, and KH/RB-CQDs/PVA; Figure S22: Mechanical properties of PVA, RB-CQDs/PVA, and KH/RB-CQDs/PVA; Figure S23: PVA, CK, and KH/RB-CQDs/PVA treated potatoes: (a) Stem height, (b) Root length, (c) Fresh stem weight, (d) Dry stem weight, (e) Dry root weight, (f) Fresh root weight; Figure S24: PVA, CK, and KH/RB-CQDs/PVA treated potatoes: (a) ETR, (b) NPQ, (c) qP, (d) Y(NPQ), (e) Y(NO), (f) Y(II); Figure S25: PVA, CK, and KH/RB-CQDs/PVA treated potatoes: (a) qN, (b) Fv/Fm, (c) Fm’, (d) qL, (e) Fo, (f) Fm; Table S1: Comparison of recently reported carbon dot-based agricultural light-conversion films (2021–2025).
