# Supplementary material for: Carbon-Core/Molecular-State-Regulated Red/Blue Dual-Emission Carbon Quantum Dots Covalently Anchored on Polyvinyl Alcohol for Multifunctional Agricultural Films in Greenhouse Potato Production
Source: Polymers (Basel). 2026 Jun 9;18(12):1442. doi: 10.3390/polym18121442 (PMC13307153; doi:10.3390/polym18121442)
Supplement: Supplementary file 1 [file polymers-18-01442-s001.zip › polymers-4345461-supplementary.pdf]

Supporting Information

Carbon-Core/Molecular-State-Regulated Red/Blue Dual-Emission Carbon Quantum Dots Covalently Anchored on Polyvinyl Alcohol for Multifunctional Agricultural Films in Greenhouse Potato Production

Zhimin Ye [1,†], Jiwei Liu [1,†], Maolin Wang [1], Kun Huang [2], Li Zhang [1], Yuanyuan Jiang [1], Ying Wang [3], Yunsong Zhang [1,*] and Li Lin [1,*,†]

[1] College of Science, Sichuan Agricultural University, Ya'an 625014, China; yzm13398308553@163.com (Z.Y.); 6jvliu@gmail.com (J.L.); maolin1217@126.com (M.W.); zhangli@sicau.edu.cn (L.Z.); yyjiang607@sina.cn (Y.J.)

[2] National Engineering Research Center of Clean Technology in Leather Industry, Sichuan University, Chengdu 610065, China; gmzc2026@163.com

[3] College of Water Conservancy and Hydropower Engineering, Sichuan Agricultural University, Ya'an 625014, China; yw1981@gmail.com

* Correspondence: yaanyunsong@sicau.edu.cn (Y.Z.); 14211@sicau.edu.cn (L.L.)

† These authors contributed equally to this work.

1. Supplementary Figures

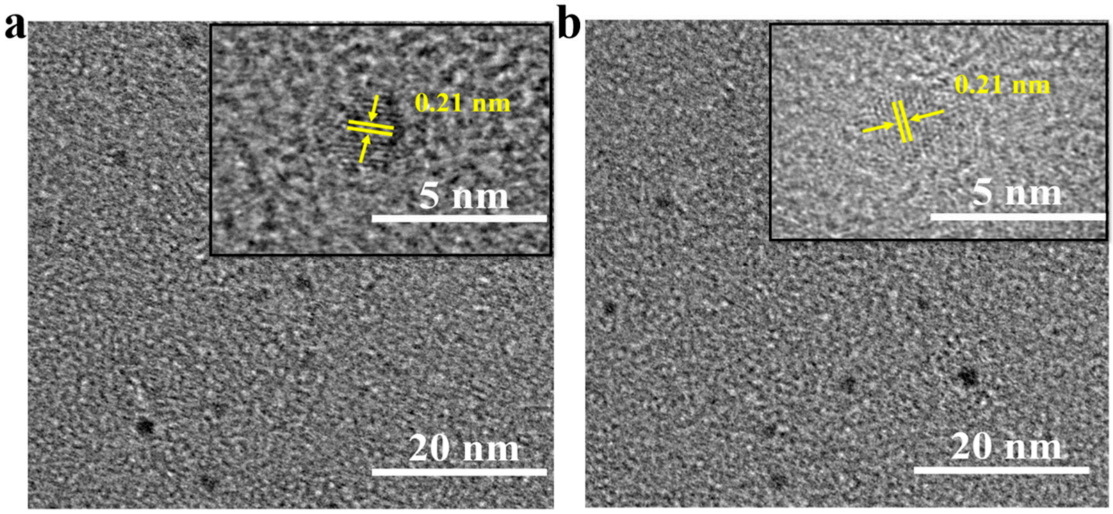

Figure S1.TEM image of (a) B-CQDs and (b) G-CQDs.

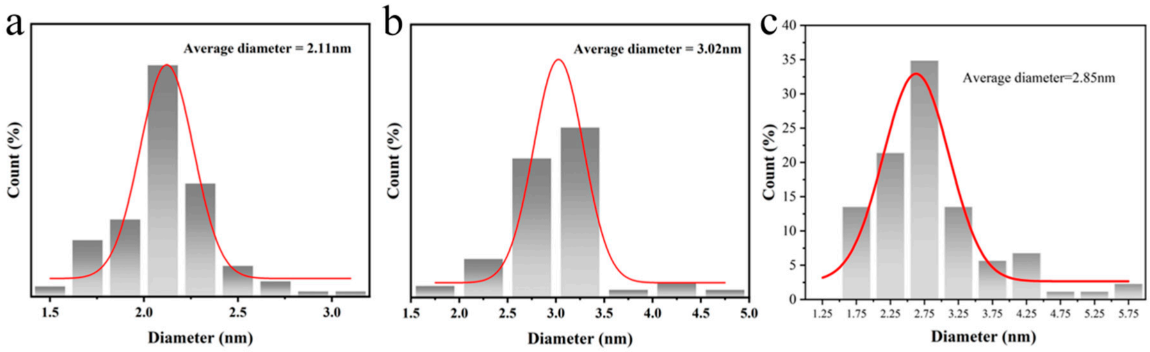

Figure S2. Size distribution histogram of (a) B-CQDs (b) G-CQDs and (c) RB-CQDs.

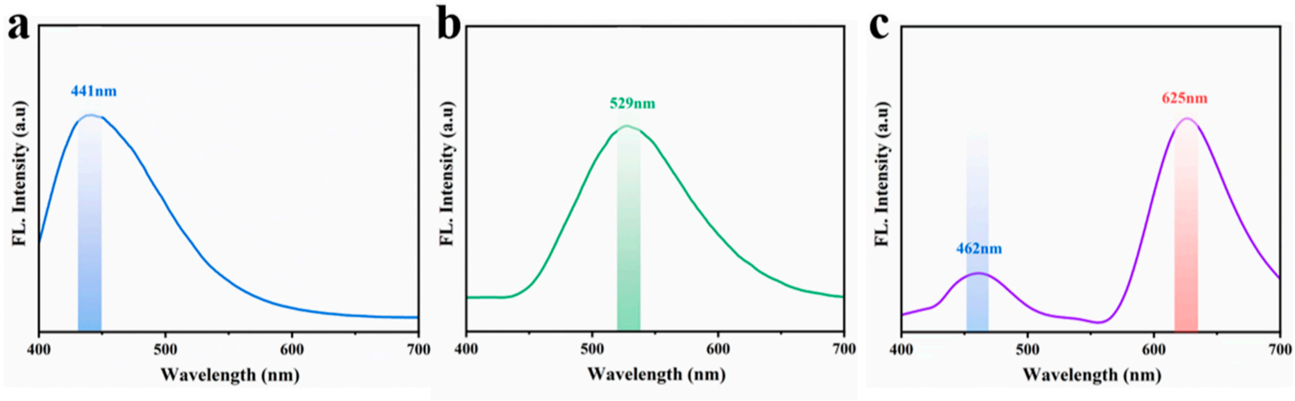

Figure S3. Fluorescence emission spectrum of (a) B-CQDs (b) G-CQDs and (c) RB-CQDs.

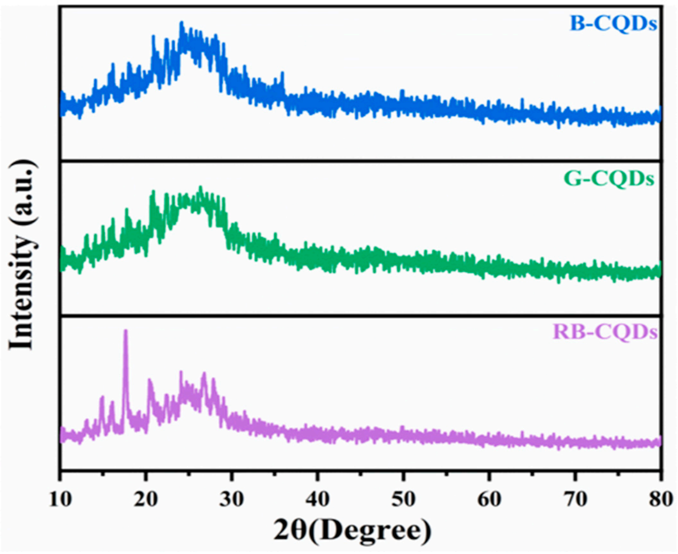

Figure S4. XRD images of B-CQDs, G-CQDs, and RB-CQDs.

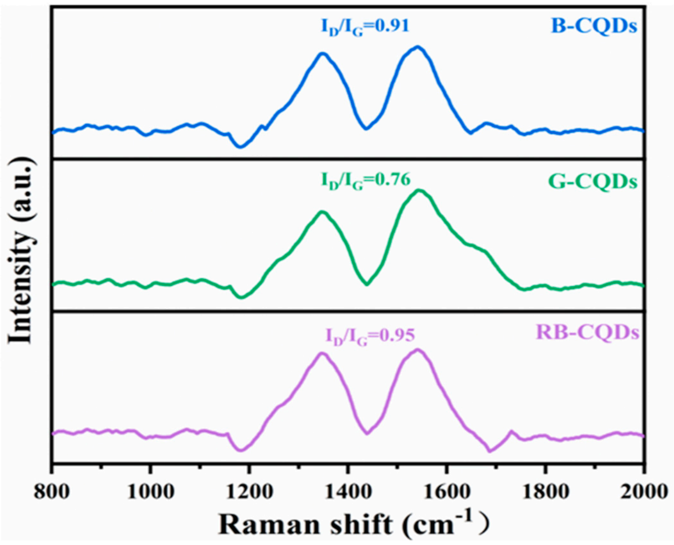

Figure S5. Raman images of B-CQDs, G-CQDs, and RB-CQDs.

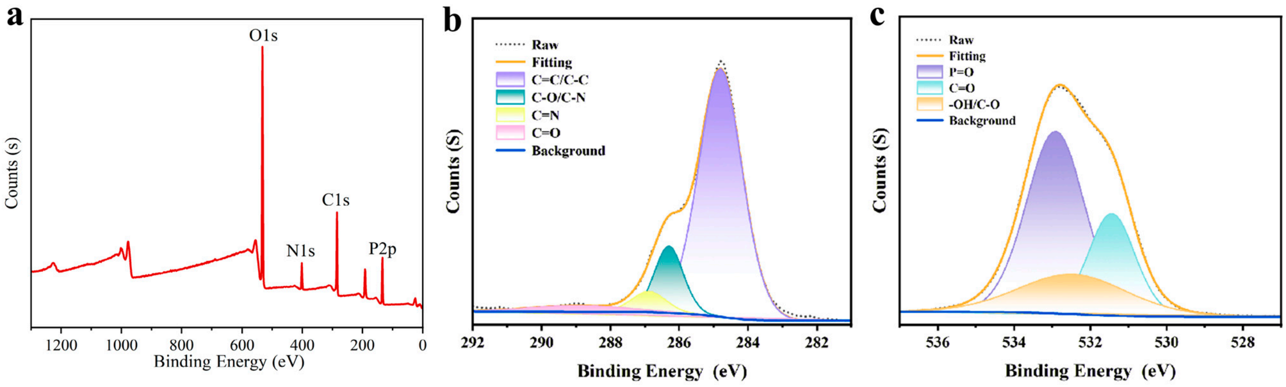

Figure S6. XPS spectra of RB-CQDs: (a) survey spectrum, (b) C 1s spectrum, (c) O 1s spectrum.

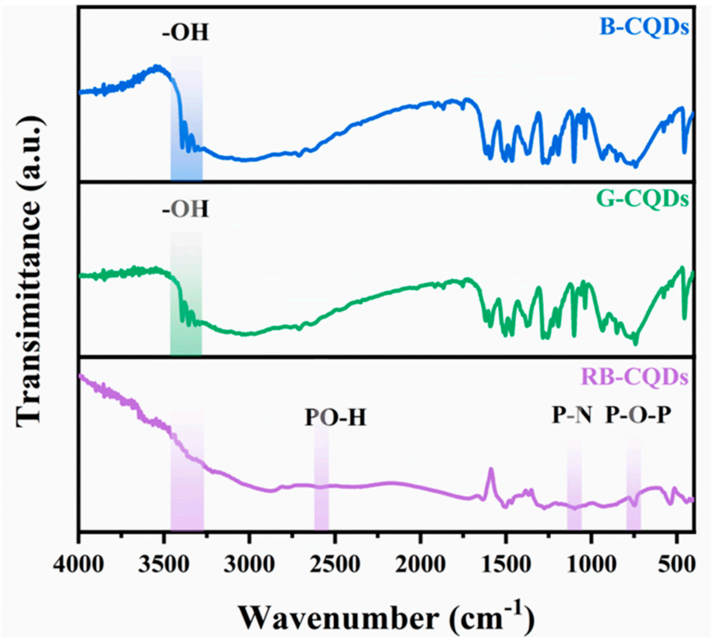

Figure S7. Infrared images of B-CQDs, G-CQDs, and RB-CQDs.

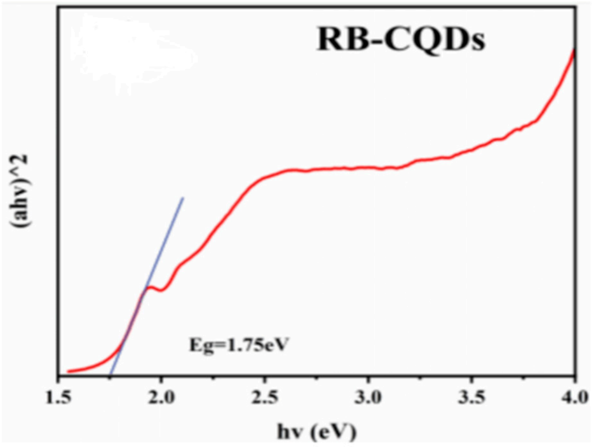

Figure S8. Bandgap energy diagram of RB-CQDs.

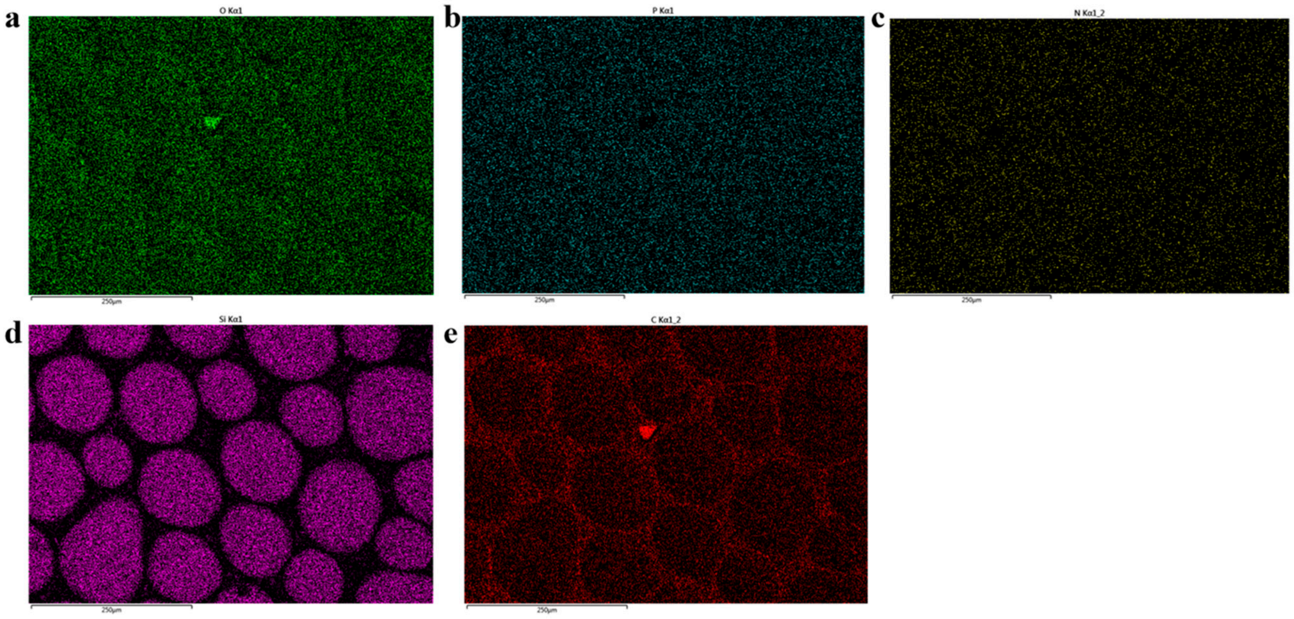

Figure S9. EDS elemental spectra of KH/RB-CQDs/PVA nanocomposite: (a) O spectrum, (b) P spectrum, (c) N spectrum, (d) Si spectrum, (e) C spectrum.

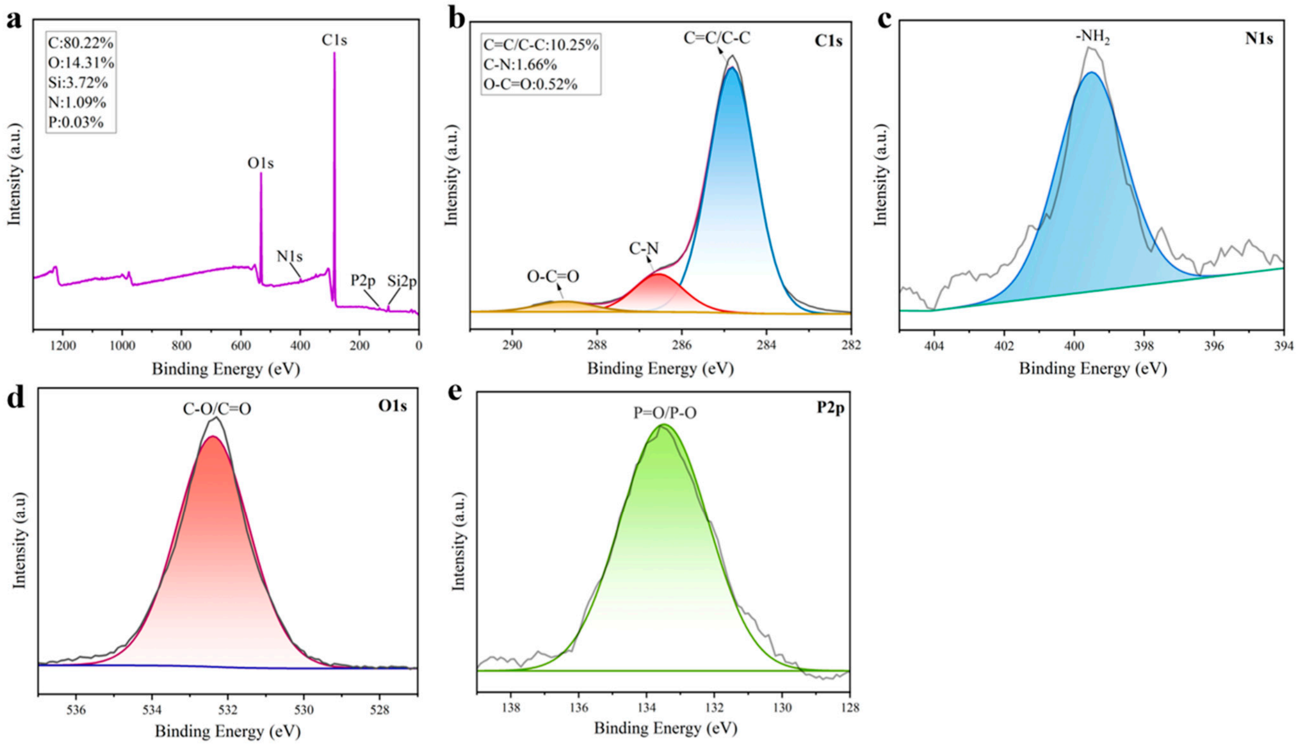

Figure S10. XPS spectra of KH/RB-CQDs/PVA nanocomposite: (a) survey spectrum, (b) C 1s spectrum, (c) N 1s spectrum, (d) O 1s spectrum, (e) P 2p spectrum.

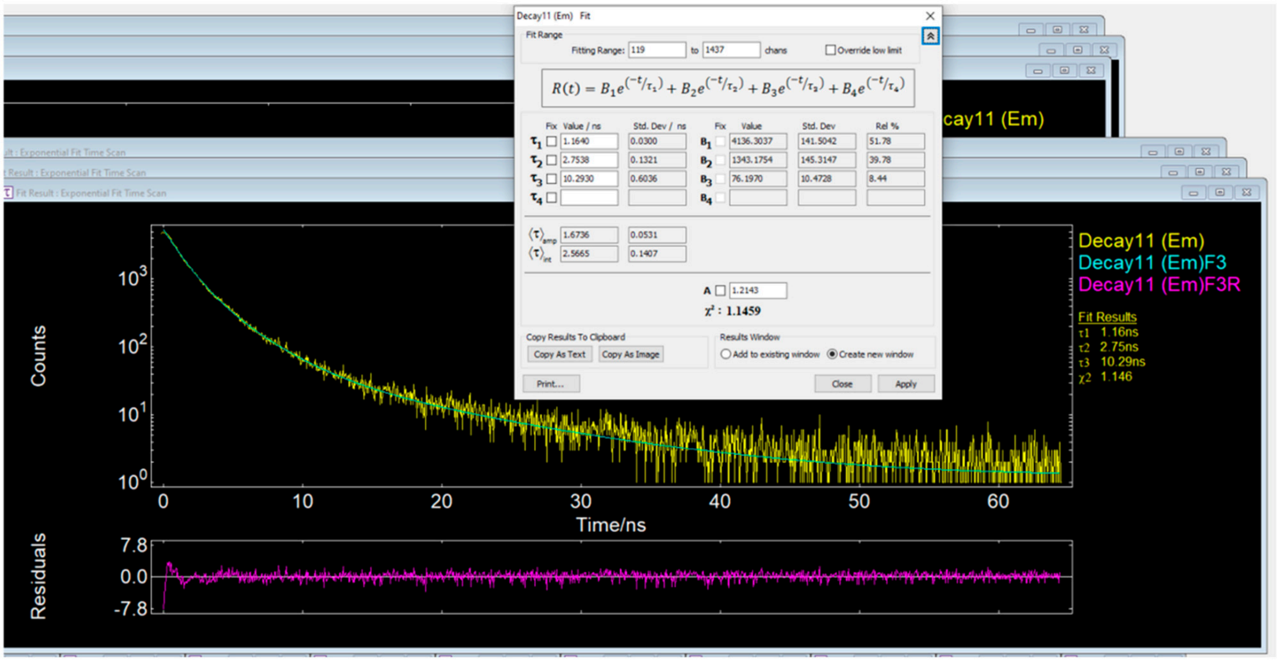

Figure S11. Fluorescence lifetime of RB-CQDs at 450 nm.

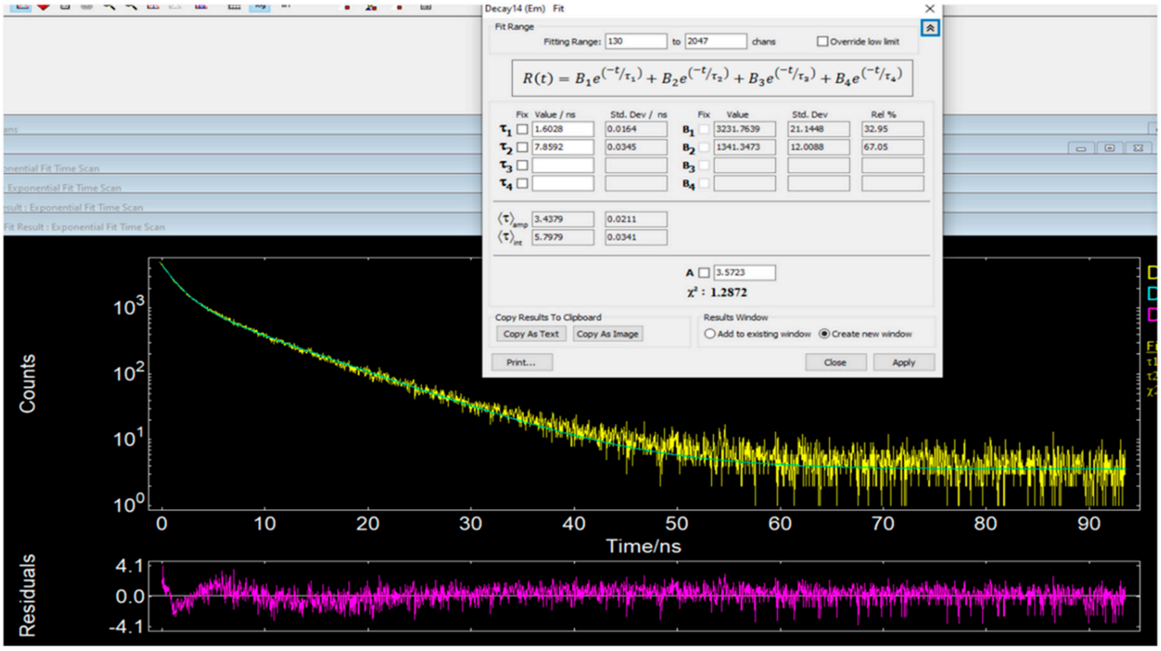

Figure S12.Fluorescence lifetime of RB-CQDs at 620 nm.

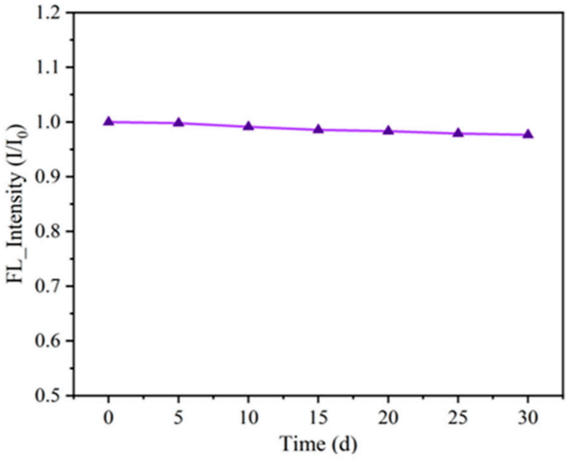

Figure S13. Long-term fluorescence stability of RB-CQDs.

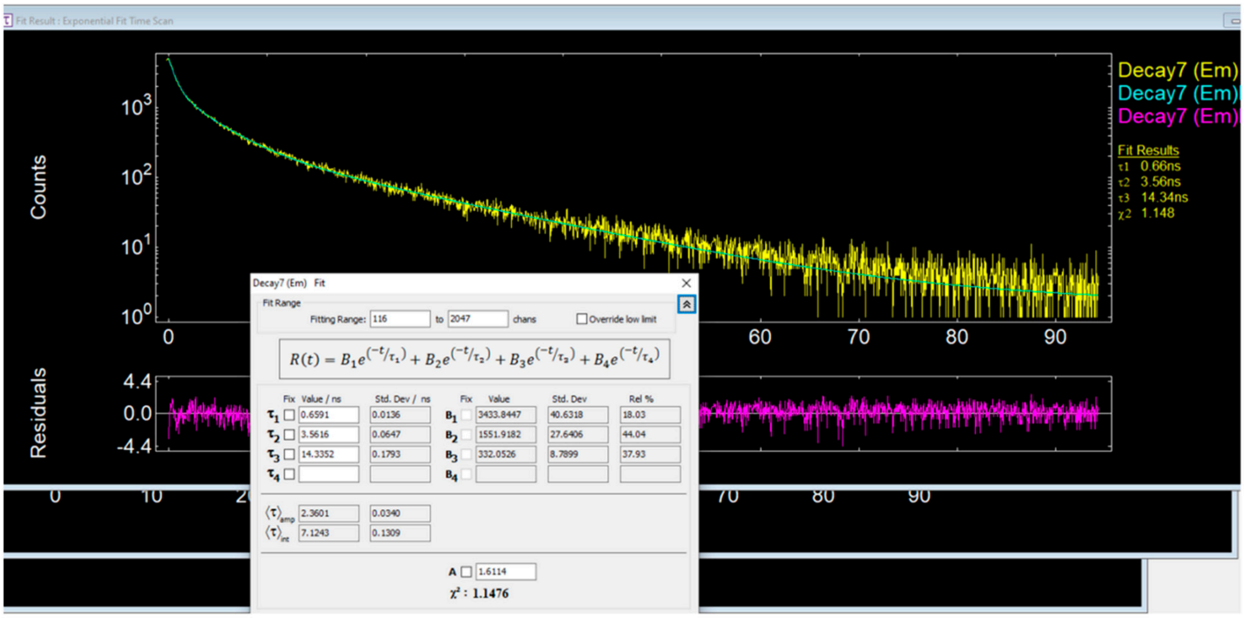

Figure S14. Fluorescence lifetime of KH/RB-CQDs/PVA at 450 nm.

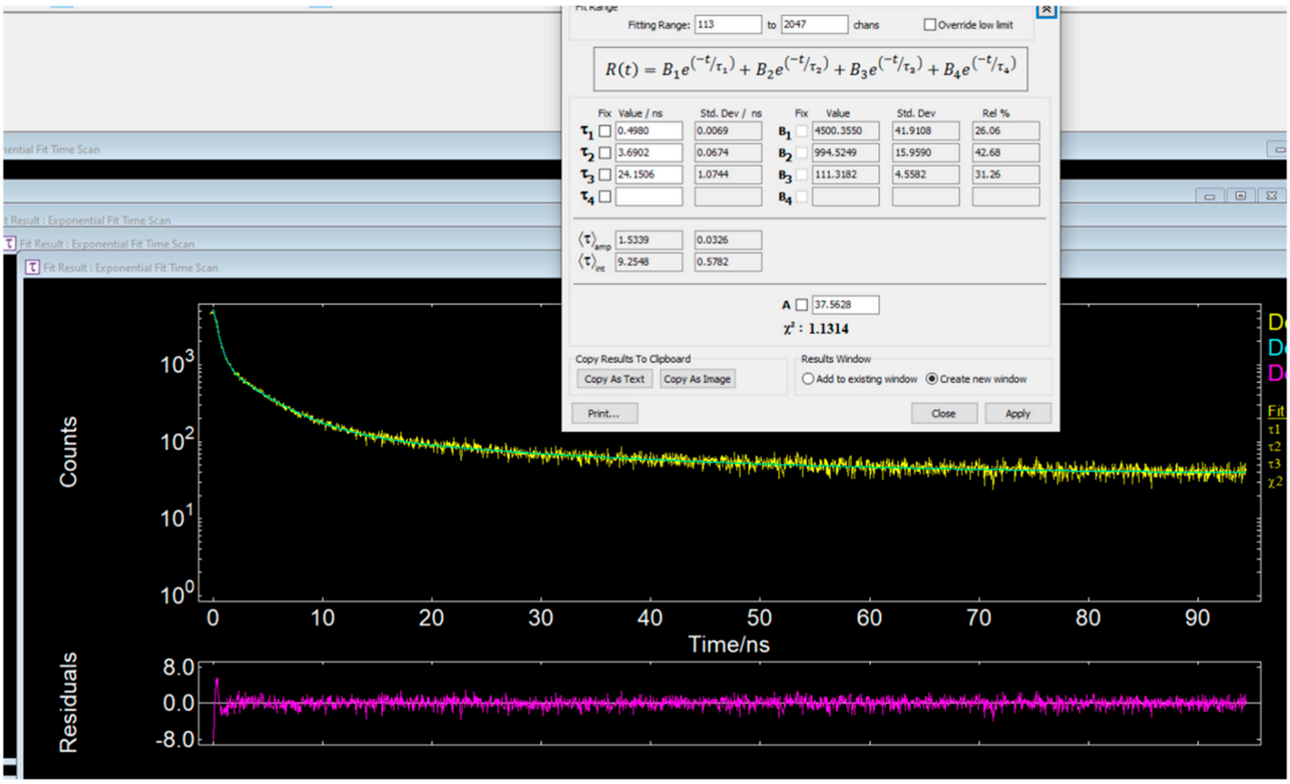

Figure S15. Fluorescence lifetime of KH/RB-CQDs/PVA at 620 nm.

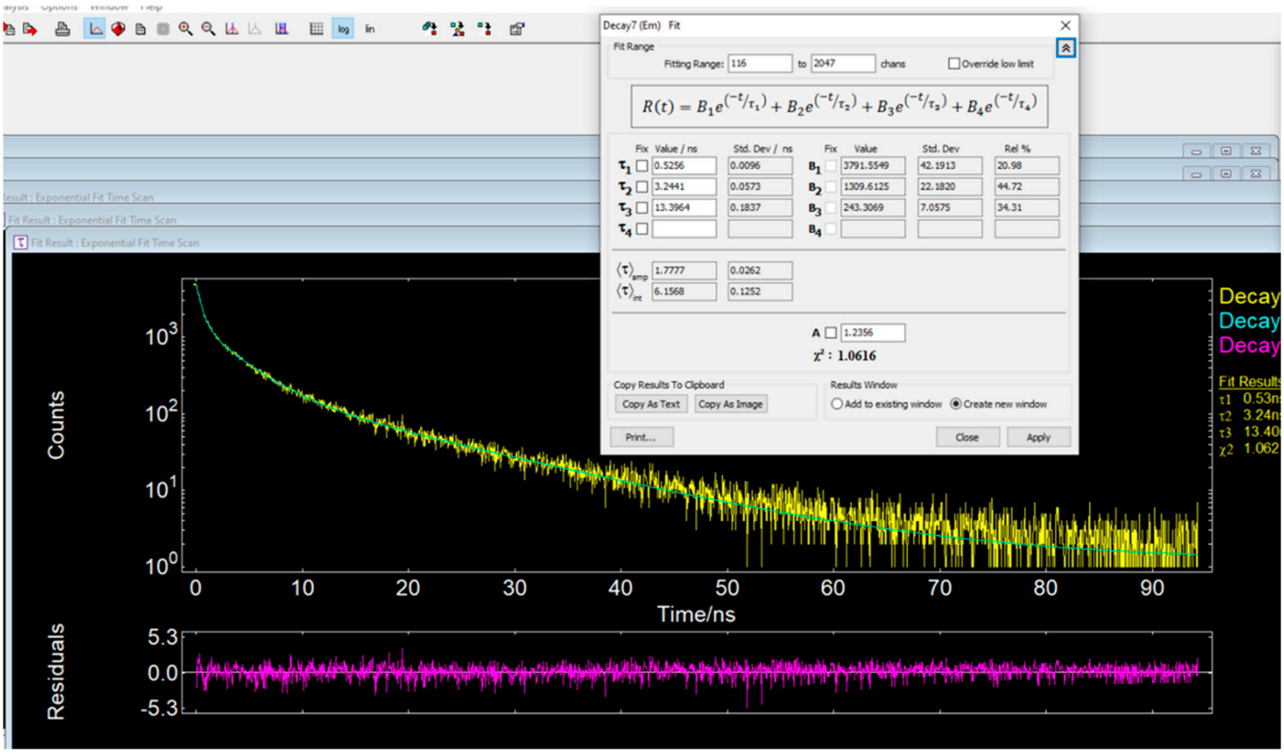

Figure S16. Fluorescence lifetime of RB-CQDs/PVA at 450 nm.

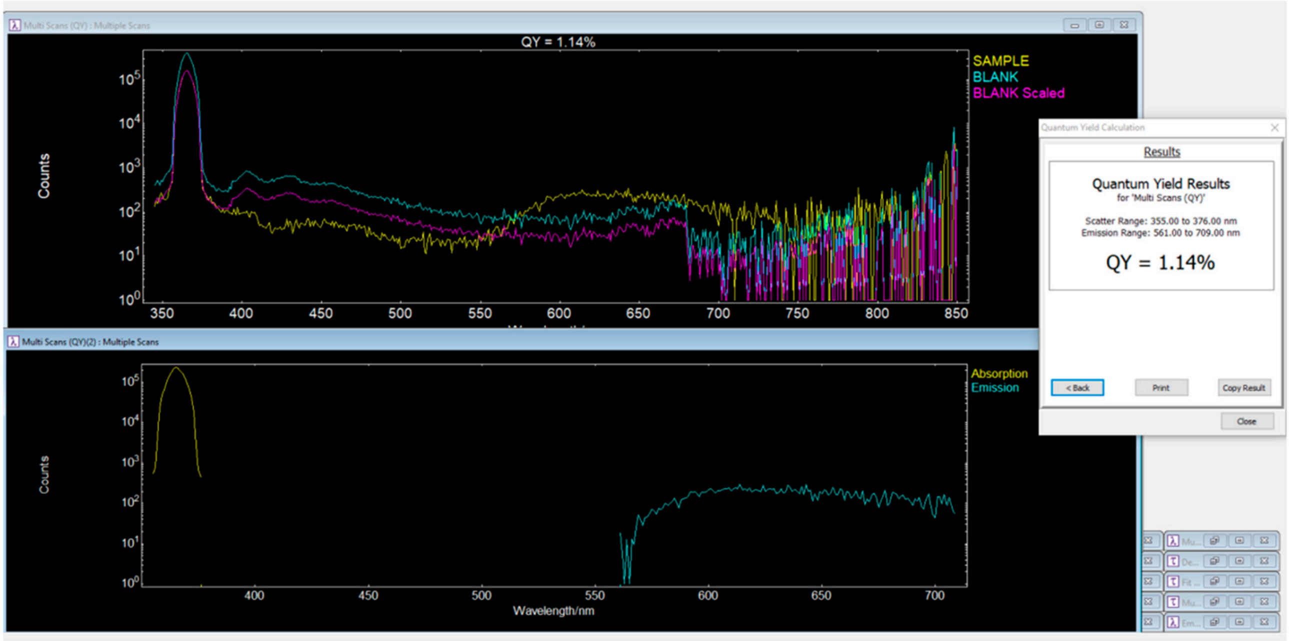

Figure S17. Fluorescence quantum yield of RB-CQDs.

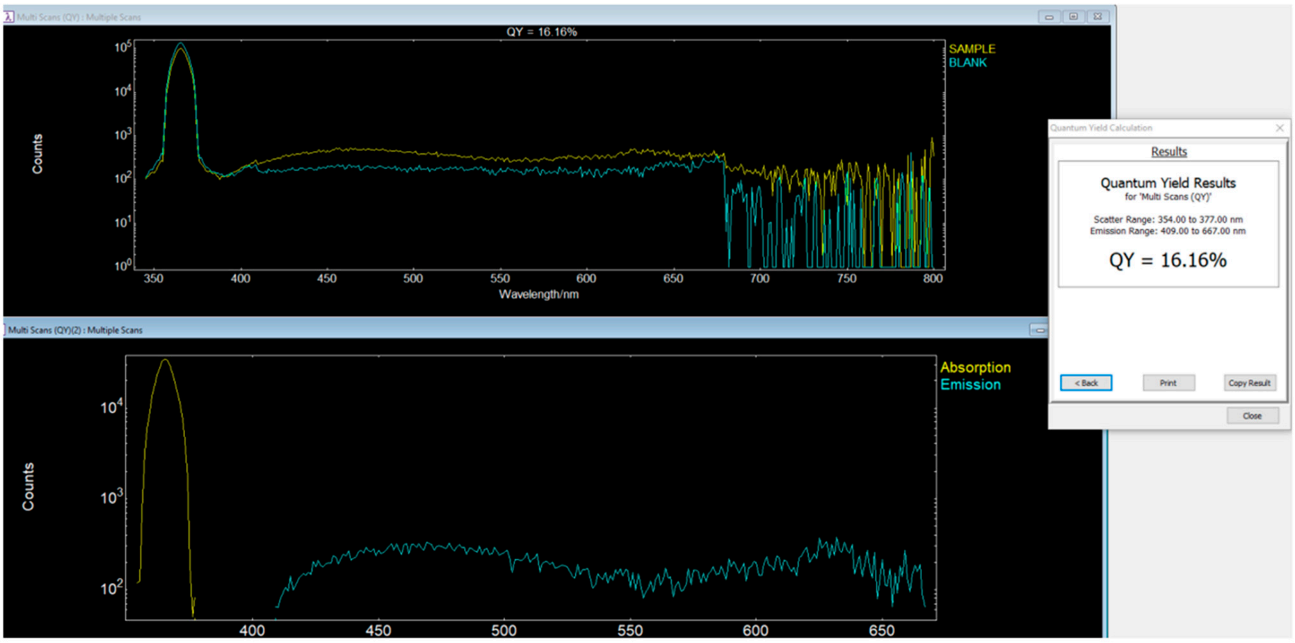

Figure S18. Fluorescence quantum yield of KH/RB-CQDs/PVA.

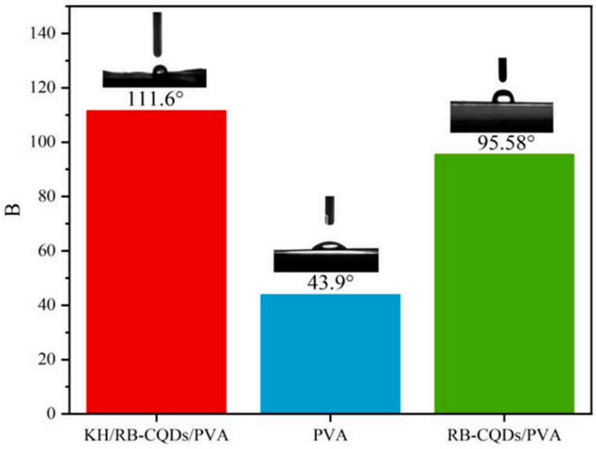

Figure S19. Water contact angles of PVA, RB-CQDs/PVA, and KH/RB-CQDs/PVA.

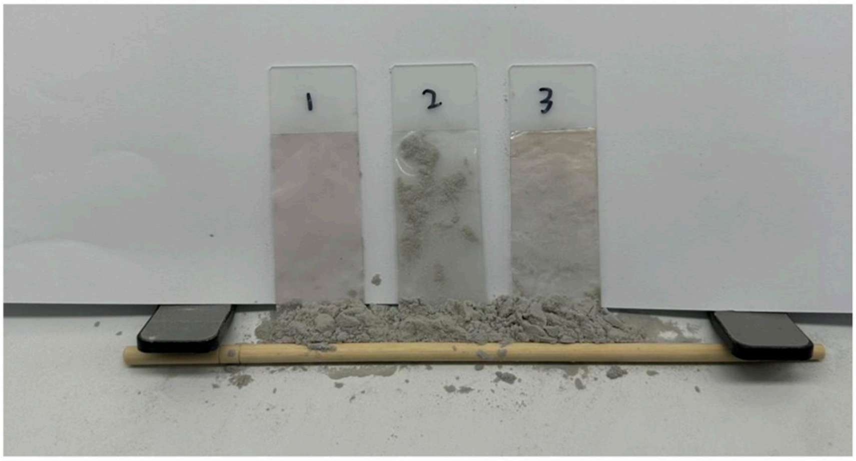

Figure S20. Dust removal images of PVA, RB-CQDs/PVA, and KH/RB-CQDs/PVA.

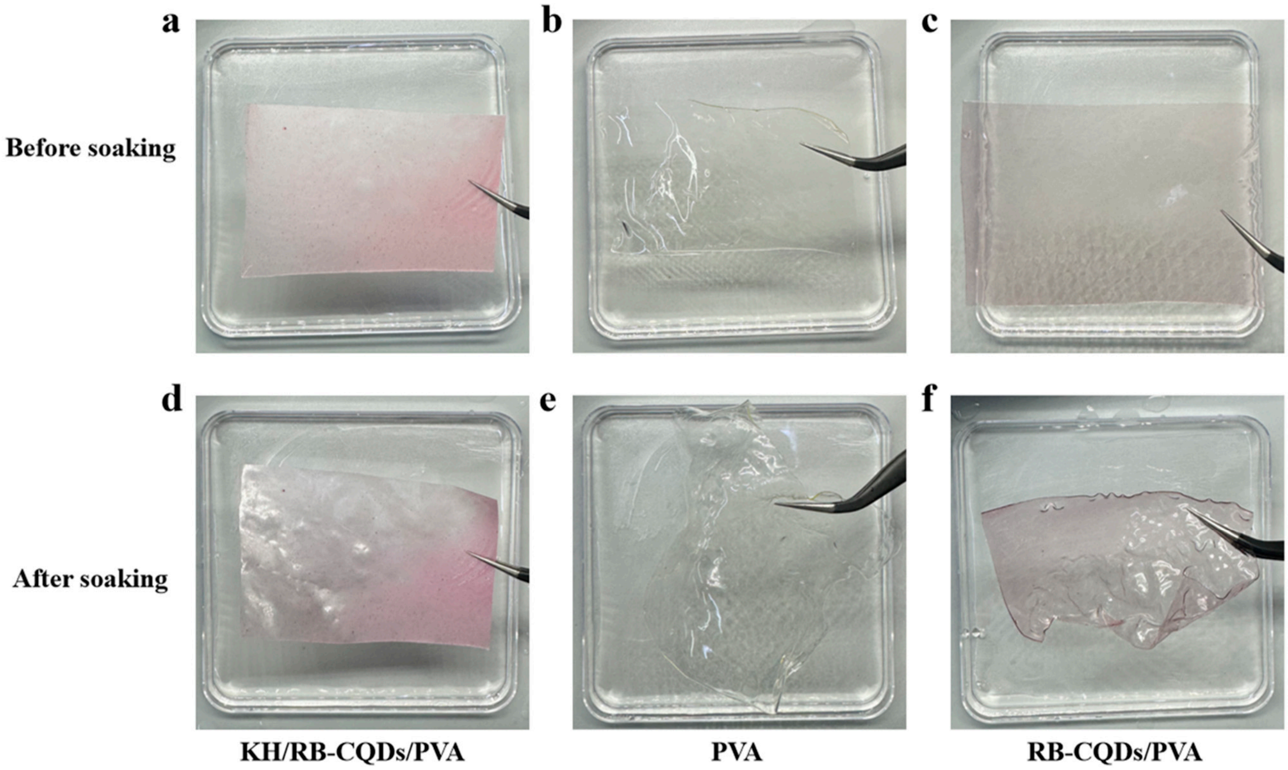

Figure S21. (a-c) Images before immersion of PVA, RB-CQDs/PVA, and KH/RB-CQDs/PVA, (d-f) Images after immersion of PVA, RB-CQDs/PVA, and KH/RB-CQDs/PVA.

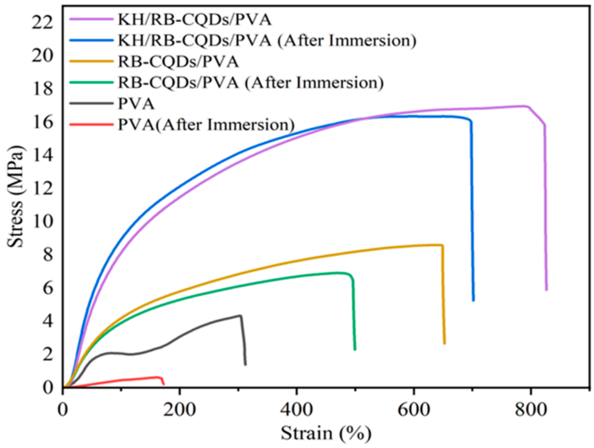

Figure S22. Mechanical properties of PVA, RB-CQDs/PVA, and KH/RB-CQDs/PVA.

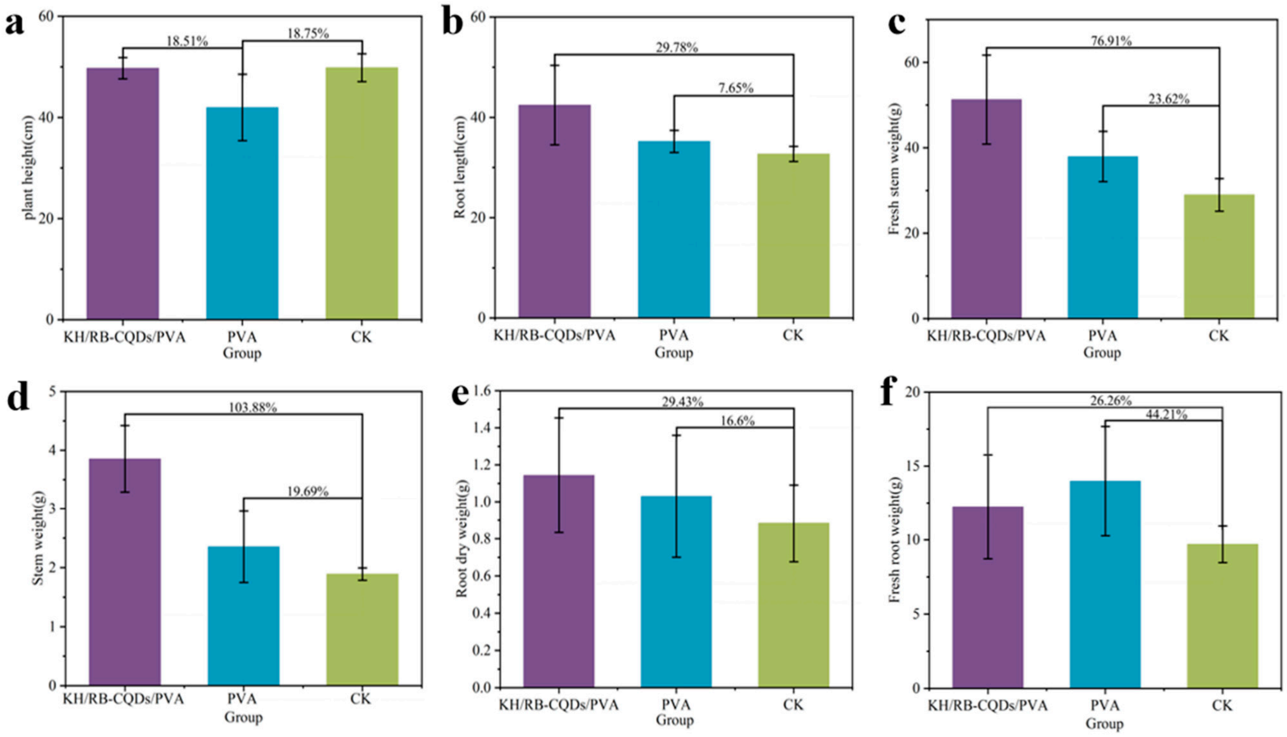

Figure S23. PVA, CK, and KH/RB-CQDs/PVA treated potatoes: (a) Stem height, (b) Root length, (c) Fresh stem weight, (d) Dry stem weight, (e) Dry root weight, (f) Fresh root weight.

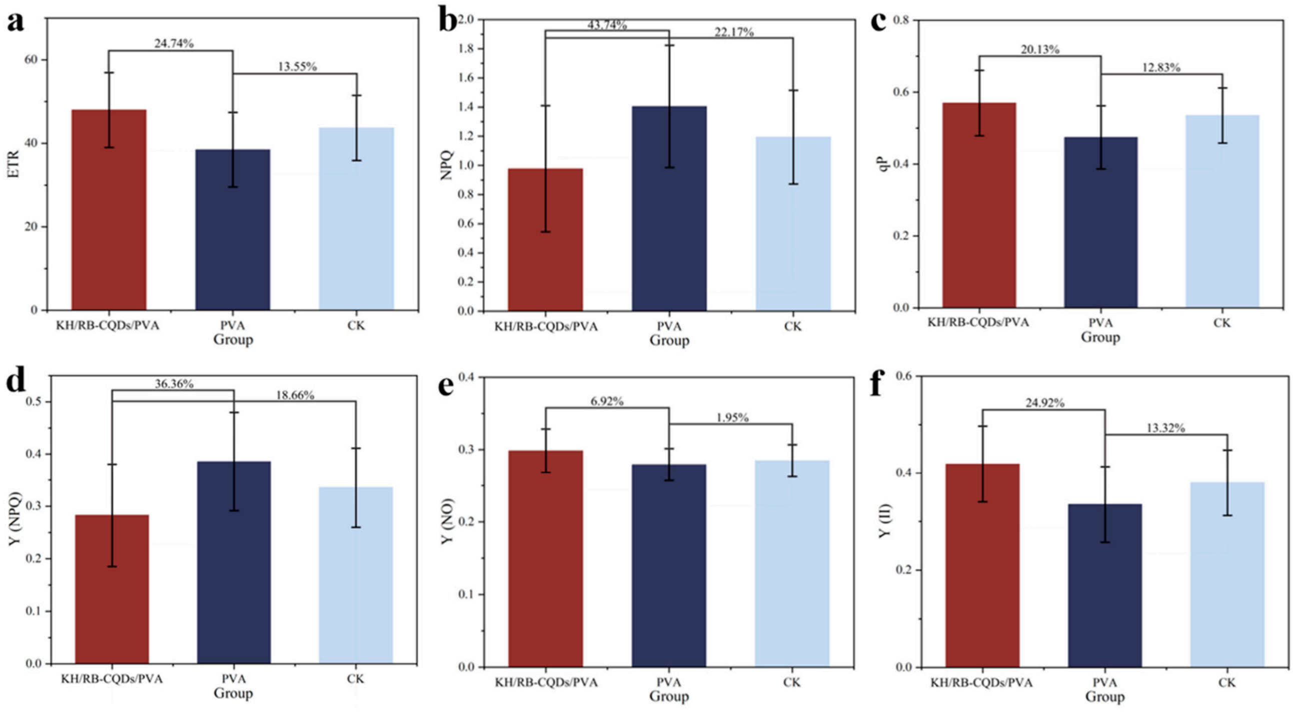

Figure S24. PVA, CK, and KH/RB-CQDs/PVA treated potatoes: (a) ETR, (b) NPQ, (c) qP, (d) Y(NPQ), (e) Y(NO), (f) Y(II)

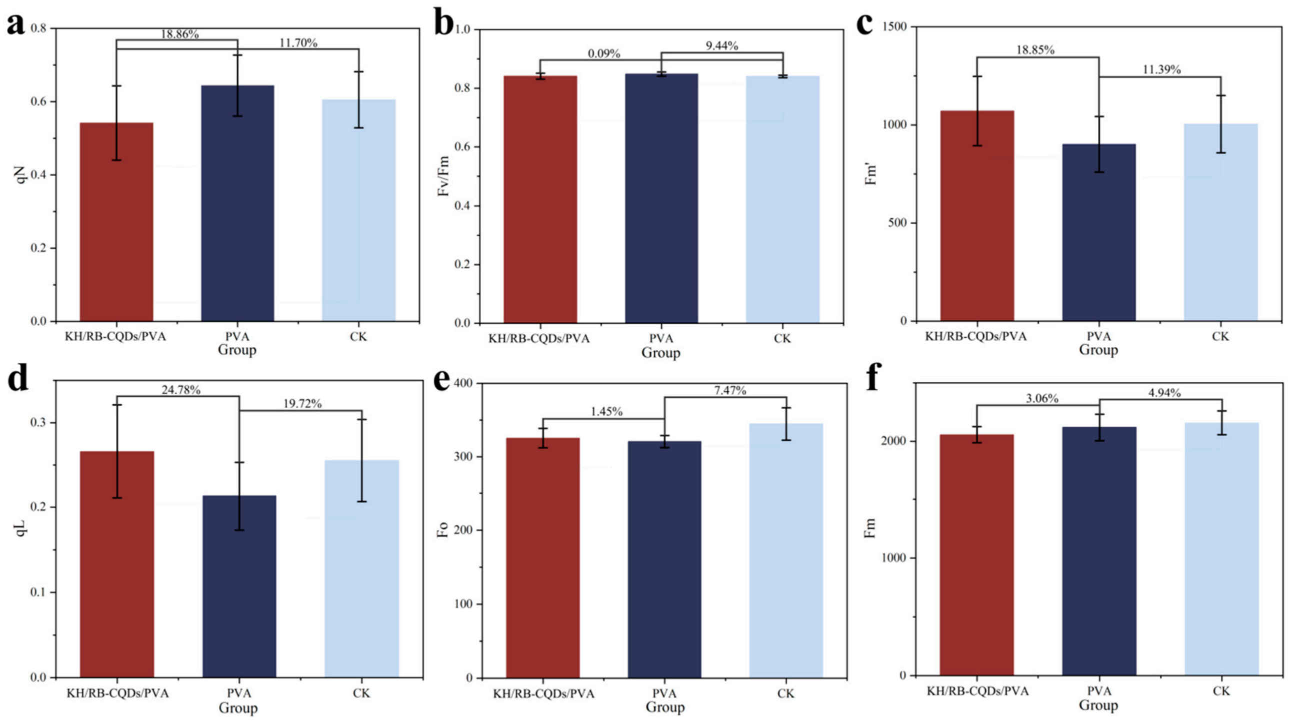

Figure S25. PVA, CK, and KH/RB-CQDs/PVA treated potatoes: (a) qN, (b) Fv/Fm, (c) Fm′, (d) qL, (e) Fo, (f) Fm

Table S1. Comparison of recently reported carbon dot-based agricultural light-conversion films (2021–2025).

Entry	Light-emitting material	Matrix	Crops	Main effect	Refs.
1	B/R-CDs	PVA film	Bok choy, lettuce, choy sum	Fresh weight +152.73% (bok choy)	[59]
2	Biomass-derived CQDs	Dual light-conversion film	Arabidopsis	Biomass +180%, CO_2 fixation +240% (cyanobacteria)	[60]
3	B&R-CDs	Regenerated cellulose + SAE coating		Transmittance 89.7%, 112-day degradation	[61]
4	RCDs	PVA film	Mung bean	Fresh weight	[26]

Entry	Light-emitting material	Matrix	Crops	Main effect	Refs.
			sprouts	+10.4%, dry weight +13.9%	
5	CDs (green-to-red)	Foliar spraying	Tomato	Plant height +10.26%, fresh weight +19.81%	[62]
6	RB-CQDs	PVA (KH-560 covalent cross-linking)	Potato	Solid-state QY 16.16%, UV shielding 95%, yield+76%, self-cleaning (111.6°)	This work

References

59. Yu, S.; Song, Y.; Yu, H.; Wang, S.; Chen, W. Preparation of high-performance blue-red dual-emission carbon dots and their application in light conversion films. *Journal of Materials Chemistry C* **2025**, *13*, 14433–14442, doi:10.1039/D5TC01374G.

60. Ge, M.; Yuan, Y.; Liu, S.; Li, J.; Yang, C.; Du, B.; Pang, Q.; Li, S.; Chen, Z. Enhancing plant photosynthesis with dual light conversion films incorporating biomass-derived carbon dots. *Carbon Capture Science & Technology* **2024**, *13*, 100253, doi:https://doi.org/10.1016/j.ccst.2024.100253.

61. Wu, C.; Zhang, S.; He, J. A transparent and waterproof cellulose composite film with carbon quantum dots: A greener agricultural film capable of light management. *International Journal of Biological Macromolecules* **2025**, *320*, 146133, doi:https://doi.org/10.1016/j.ijbiomac.2025.146133.

26. Li, D.; Kou, E.; Li, W.; Zhang, H.; Zhang, X.; Zhuang, J.; Liu, Y.; Hu, C.; Zheng, Y.; Yang, Q.; et al. Oxidation-induced quenching mechanism of ultrabright red carbon dots and application in antioxidant RCDs/PVA film. *Chemical Engineering Journal* **2021**, *425*, 131653, doi:https://doi.org/10.1016/j.cej.2021.131653.

62. Li, S.; Zhao, W.; Zhao, W.; Jiao, Z.; Cui, K.; Wang, R.; Wang, A.; Tan, Y.; Li, H.; Yin, Y.; et al. Carbon dots promote tomato growth and yield via photosynthesis enhancement and leaf senescence delay. *Journal of Plant Physiology* **2025**, *313*, 154616, doi:https://doi.org/10.1016/j.jplph.2025.154616.
